# Utility elicitation in adults and children for allergic rhinoconjunctivitis and associated health states

**DOI:** 10.1007/s11136-018-1910-8

**Published:** 2018-06-08

**Authors:** Jenny Retzler, Tobias Sydendal Grand, Anne Domdey, Adam Smith, Mercedes Romano Rodriguez

**Affiliations:** 1 York Health Economics Consortium, Enterprise House, University of York, Innovation Way, York, YO10 5NQ UK; 2 ALK Abelló, Horsholm, Denmark

**Keywords:** Utility elicitation, Standard gamble, Visual analogue scale, Allergic rhinoconjunctivitis, Asthma, Children

## Abstract

**Purpose:**

Allergic rhinitis and asthma symptoms are detrimental to health-related quality of life (HRQoL). Health technology appraisal agencies often require cost–utility analysis when assessing new interventions. Appropriate utility estimates, which quantify the value of different conditions in cost–utility analyses, are scarce for allergic rhinitis and asthma health states. This study aimed to generate utilities for allergic rhinitis and asthma health states from a European general population sample of adults and children.

**Methods:**

Health state descriptions incorporating symptoms, impact of symptoms on daily life and symptom treatment were developed using clinical guidelines. Descriptions were amended with clinician and patient input, and incorporated into a survey in which each health state was followed by a standard gamble (adults) or visual analogue scale (children) item. The survey was distributed to samples of adults and children aged 8 to 11 from four European countries that were stratified to represent the general population within that country.

**Results:**

1454 adults and 1082 children completed the survey. Mean health utilities ranged from 0.635 to 0.880 and those elicited in children were lower (0.635 to 0.705) than those elicited in adults (0.812 to 0.880). Disutilities assessing the impact of increased allergic rhinitis severity and comorbidities were also greater in children than in adults.

**Conclusions:**

Symptoms of allergic rhinitis and asthma were valued as having a clinically meaningful impact on HRQoL. Children valued health states as poorer than adults, and further research should investigate whether this reflects true preferential differences or results from methodological and/or comprehension differences between the two groups.

**Electronic supplementary material:**

The online version of this article (10.1007/s11136-018-1910-8) contains supplementary material, which is available to authorized users.

## Introduction

### Background

Allergic rhinitis (AR) is a chronic inflammatory condition affecting the upper airways, caused by an excess of immunoglobin E produced in response to environmental allergens such as house dust mites and pollen. AR affects 17–26% of the population in Europe, with prevalence expected to rise [1]. While the symptoms of nasal blockage, sneezing and itching [2] may be thought of as trivial, research shows they have a detrimental impact on health-related quality of life (HRQoL), disturbing sleep, concentration and productivity at work or school, and the ability to conduct daily activities and causing widespread discomfort [3, 4].

The persistence and nature of symptoms experienced can vary depending on the allergen(s) responsible and the pattern of exposure. When AR is triggered by pollen, ocular inflammation can occur alongside nasal symptoms, broadening the condition to allergic rhinoconjunctivitis (ARC), commonly known as ‘hay fever’. Individuals may respond to multiple allergens, and AR/ARC is further complicated by commonly presenting with asthma, a chronic inflammatory condition of the lower airways that causes coughing, wheezing, chest tightness and breathlessness [5].

AR/ARC is found in over 80% of asthma patients, and asthma is found in 20–60% of those with AR/ARC [6], leading researchers to hypothesise that AR/ARC and asthma are manifestations of the same disease, affecting different parts of a ‘united airway’ [7]. Moreover, AR/ARC often precedes asthma onset, and has been shown to be one of the strongest independent risk factors for asthma development [6], with evidence that AR/ARC can increase the risk of adult-onset asthma threefold [8] and that AR severity can impact asthma symptom control [6]. Once developed, asthma commonly requires lifelong treatment [5], with comorbid disease impacting HRQoL to a greater extent than AR or asthma alone, particularly detrimental to physical functioning [3, 9].

Recommended first-line treatments for AR/ARC are usually allergen avoidance and pharmacological symptom management [1, 6]. It can be difficult to avoid airborne allergens like pollen and house dust mites, however, and symptom-relieving medications, commonly anti-histamine or corticosteroid based, do not target the cause of the disease, and are therefore unlikely to prevent asthma onset [10]. For some patients, these treatment options are ineffective, and even where symptoms are managed successfully, patients are required to maintain daily treatment indefinitely. Allergy immunotherapy (AIT) is a treatment option available to patients with AR/ARC whose symptoms are not adequately controlled by symptom-relieving medication. AIT aims to desensitise the immune response to trigger allergens, thereby treating the underlying disease. Evidence indicates that AIT in childhood can reduce the risk of developing comorbid asthma 7 years after treatment [10].

Health technology appraisal agencies increasingly stipulate cost–utility analysis (CUA) as a part of the assessment of new technologies. CUA compares the incremental costs and health benefits of two or more interventions. Health benefits are measured as health utilities, allowing for standardised comparison across different disease areas. Health utilities represent the value a particular population places on the impact of a health condition on HRQoL, thereby combining HRQoL with the ‘desirability’ of a condition. Correspondingly, disutility can also be measured. Disutility represents the decrement in utility due to a particular symptom or complication, and is calculated by measuring the difference between two health states that are identical in all aspects aside from the complication or symptoms of interest [11].

Utility inputs for the different combinations of seasonal ARC and associated health states that may be required for CUA are scarce, and, to our knowledge, non-existent in paediatric populations. This study aimed to generate a set of utilities and disutilities from four European countries for seasonal ARC and associated conditions, in both adult and paediatric populations, for use in future cost–utility analyses.

## Methods

### Ethical approval

This study was conducted in accordance with the Declaration of Helsinki and ethical approval was granted by the University of York Health Sciences research ethics committee. Informed parental consent was provided for all child respondents.

### Health states

Fourteen health states (Table 1) were defined that represented three levels of severity in ARC (mild, moderate and severe), during and outside pollen season, in combination with comorbidities of asthma, and allergic rhinitis triggered by perennial allergens. Vignettes describing these states were developed using current clinical guidelines (Allergic Rhinitis and its Impact on Asthma (ARIA) [12], Global Initiative of Asthma (GINA) [13]) to incorporate (i) symptoms, (ii) how symptoms impact daily life and (iii) commonly required treatments.

**Table 1:** Health states

During the Pollen season					Health state for elicitation
Diagnosis		ARC severity (seasonal allergies)	Severity of comorbid asthma	Presence of comorbid perennial allergic rhinitis?	
1	Seasonal ARC	Mild	–	n/a^a^	Mild ARC
2	Seasonal ARC	Moderate	–	n/a^a^	Moderate ARC
3	Seasonal ARC	Severe	–	n/a^a^	Severe ARC
4	Seasonal ARC with asthma	Mild	Well-to-partly controlled	n/a^a^	Mild ARC + well-to-partly controlled asthma
5	Seasonal ARC with asthma	Mild	Uncontrolled	n/a^a^	Mild ARC + uncontrolled asthma
6	Seasonal ARC with asthma	Moderate	Well-to-partly controlled	n/a^a^	Moderate ARC + well-to-partly controlled asthma
7	Seasonal ARC with asthma	Moderate	Uncontrolled	n/a^a^	Moderate ARC + uncontrolled asthma
8	Seasonal ARC with asthma	Severe	Well-to-partly controlled	n/a^a^	Severe ARC + well-to-partly controlled asthma
9	Seasonal ARC with asthma	Severe	Uncontrolled	n/a^a^	Severe ARC + uncontrolled asthma

Outside the Pollen season					Health state for elicitation
Diagnosis		ARC severity (seasonal allergies)b	Severity of comorbid asthma	Presence of comorbid perennial allergic rhinitis?	
10	Seasonal ARC and perennial AR	–	–	Yes	Perennial AR
11	Seasonal ARC with asthma	–	Well-to-partly controlled	–	Well-to-partly controlled asthma
12	Seasonal ARC with asthma	–	Uncontrolled	–	Uncontrolled asthma
13	Seasonal ARC and perennial AR with asthma	–	Well-to-partly controlled	Yes	Perennial AR + well-to-partly controlled asthma
14	Seasonal ARC and perennial AR with asthma	–	Uncontrolled	Yes	Perennial AR + uncontrolled asthma

^a^Assumes that during the pollen season, comorbid perennial allergy symptoms are combined with ARC symptoms and do not require additional health states

^b^Assumes that utility would be normal in patients with seasonal ARC and no comorbidities regardless of severity

Two expert clinicians (one paediatric specialist) provided written feedback on the vignettes. Revised vignettes were sent to eight patients with seasonal ARC and the associated health conditions (four patients with seasonal and/or perennial ARC/AR and four patients with seasonal and/or perennial ARC/AR and asthma), for a second review. Final vignettes were developed incorporating all feedback.

### Elicitation methods

The study task differed for the adult and child surveys. A standard gamble (SG) task was designed for the adult survey. SG tasks are the only technique that truly reflects decisions under uncertainty, thereby mimicking the circumstances under which we make health decisions in real life and meeting assumptions of expected utility theory. Due to comprehension issues within the child sample age range [14] and the use of a comparator of ‘death’, the SG was not considered appropriate for the child survey. Instead, a visual analogue scale (VAS) was designed for the child study.

SG tasks present respondents with a health state description, asking them to imagine they experience the symptoms described. Respondents must then decide at what probability they would decide in favour of a risky treatment in order to be cured, with a risk of death should the treatment fail, or whether, instead, to remain in the certain health state (i.e. unwilling to go ahead with risky treatment, instead preferring to continue to experience all the symptoms described). The probability of treatment success vs. failure that respondents would be willing to accept corresponds to the health utility. For each health state, SG items were developed, asking respondents to indicate their minimum ‘acceptable’ probability using a slider.

Vignettes designed for adults were adapted for lower reading ages, describing the symptoms that, for example, ‘Alex’ was feeling, to make them concrete. A VAS question and scale followed, asking children to estimate how well they thought that ‘Alex’ was feeling on a scale of 0–100, where 0 is the worst they think anyone could possibly feel, and 100 is the best they think anyone could possibly feel. They were presented with a graphical slider with images of happy and sad faces at the relevant ends for context.

### Survey

Surveys were developed using the Qualtrics online survey platform. Part one recorded demographic information and the remaining items comprised 8 of the 14 SG/VAS items per respondent (to maintain an acceptable respondent burden), randomised with even presentation using the in-built algorithm on the Qualtrics platform. The surveys took approximately 10 min to complete.

Following finalisation of the UK version of the surveys, survey text was translated by specialist health translators (TransPerfect Translations) into French, Slovakian and German. This process comprised forward and backward translations, followed by resolution processes to reconcile any differences and ensure conceptual equivalence in all languages. Translations were migrated to the online platform, where data were collected into a single database for adults, and a single database for children, regardless of the language of completion.

The sample was recruited by a third party (Qualtrics) which maintains online panel respondents who are invited to participate in online surveys in exchange for electronic points. The adult sample was selected to be broadly representative of the adult general population in each of the four countries (UK, France, Germany and Slovakia). Residents of these countries over the age of 18 years with and without ARC and associated conditions were eligible. To achieve a representative sample, quotas were set based on age and gender (Table 2). When these quotas were full, respondents meeting criteria for full quotas were no longer eligible. Recruitment was ongoing until 350 complete responses were received per country, ensuring at least 200 responses per health state. The child sample comprised children aged 8–11 years, with the upper limit reflecting the age at which child-specific drug labelling changes (12 years), and the lower limit based on evidence on the minimum age (8 years) for task comprehension [14]. Due to the narrow age range, the sample was stratified by gender only. Residents in the UK, France, Germany or Slovakia aged 8–11 were eligible. As with the adult sample, quotas were used to achieve sample stratification, and respondents meeting criteria for full quotas were no longer eligible (Table 2). Recruitment was ongoing until 260 (fewer than adults due to difficulty reaching respondents in this age group) complete responses were received per country, ensuring at least 150 responses per health state.

**Table 2:** Sample characteristics

Demographic	Adult	Child	Target^a^ (Where applicable)
Age group			
8–11	–	1082 (100%)	–
18–24	156 (10.7%)	–	10.5%
25–34	248 (17.1%)	–	16.8%
35–44	250 (17.2%)	–	17.0%
45–54	260 (17.9%)	–	17.8%
55–64	232 (16.0%)	–	15.8%
65 and over	308 (21.2%)	–	22.3%
Gender			
Male	708 (48.7%)	523 (48.3%)	48.7%
Female	746 (51.3%)	559 (51.7%)	51.3%
Self-reported relevant diagnoses			
ARC	500 (34.4%)	596 (55.1%)	–
AR	327(22.5%)	422 (39.0%)	–
Asthma	259 (17.8%)	257 (23.8%)	–

^a^Targets represent averaged country-specific demographic weightings derived from Eurostat 2015 data. Full country-by-country breakdowns are available in Supplementary Material SA. These were used for quota implementation during recruitment

Following email invitation, respondents completed the survey online. Survey invites for the children were sent to parents to ensure informed parental consent. Adult respondents and consenting parents of child respondents completed the pre-screening demographic questions to ensure that only those who met eligibility criteria were directed to complete the full survey.

### Statistical analysis

Data were exported into SPSS (v24.0) for statistical analysis. Descriptive statistics detailing the socio-demographic data for the sample (e.g. gender, age, employment status) were generated.

Extreme values, or those that lacked face validity, were removed where appropriate. All exclusion rules applied are described at each analysis. Average values (mean and median) for utilities and disutilities were calculated from the respondent-level data. Analyses of variance (ANOVAs) were conducted to investigate differences between utilities and disutilities generated by patient and non-patient populations for the relevant health states (i.e. those involving only that patient group). ‘Patients’ were defined as any respondent who self-reported either current or previous diagnosis of asthma, allergic rhinitis or hay fever. Where violations of ANOVA assumptions were identified, appropriate alternatives are reported (e.g. Brown–Forsythe *F* statistics).

## Results

### Study population

A total of 1454 adults (UK: 362, France: 368, Germany: 359, Slovakia: 365) and 1082 children (UK: 263, France: 273, Slovakia: 273, Germany: 273) completed the survey. For each country, the age and gender sample stratifications broadly met the target criteria to generate samples representative of the general population in each country (see Supplementary Material for target and actual demographics per country). Table 2 shows that there were high levels of adults self-reporting previously experiencing ARC, with fewer respondents self-reporting other diagnoses. Rates of relevant diagnoses were higher still in children, with 55% parents reporting that their child had experienced ARC.

### Utilities

Examination of box-and-whisker plots indicated that adult utilities lower than 0.3 were statistical outliers (> 1.5 interquartile ranges below the lower quartile). These values were also considered to be lacking face validity, and indicative of respondents not responding in a thoughtful manner or having misunderstood the question. The child data included greater numbers of low utility values, and those lower than 0.3 were not outliers according to the interquartile range (IQR) rule (greater than 1.5 × IQR above the upper or below the lower quartile); however, values below this level were still considered to be lacking face validity and thus, for both datasets, responses for individuals who produced ratings lower than 0.3 for any health state were removed. This resulted in inclusion of 1235 adults (exclusions per country: UK: 54; France: 55; Germany: 53; Slovakia: 57) and 423 children (exclusions per country: UK: 133; France: 151; Germany: 166; Slovakia: 209). The impact of different exclusion criteria on sample size and utility estimates was examined in sensitivity analyses (see Supplementary Material B). Average utilities obtained are shown in Table 3.

**Table 3:** Average adult and child utilities

No.	Health state	Utilities					
		Adult			Child		
		*N*	Mean (SE)	Median (IQR)	*N*	Mean (SE)	Median (IQR)
1	Well-to-partly controlled asthma	709	0.874 (0.006)	0.949 (0.800–0.993)	232	0.693 (0.011)	0.700 (0.560–0.820)
2	Uncontrolled asthma	710	0.829 (0.006)	0.884 (0.737–0.952)	227	0.635 (0.012)	0.620 (0.500–0.770)
3	Mild ARC	714	0.880 (0.006)	0.967 (0.804–0.996)	252	0.705 (0.011)	0.700 (0.590–0.850)
4	Mild ARC + well-to-partly controlled asthma	700	0.872 (0.006)	0.947 (0.800–0.993)	253	0.677 (0.010)	0.690 (0.540–0.790)
5	Mild ARC + uncontrolled asthma	714	0.844 (0.006)	0.900 (0.754–0.980)	250	0.643 (0.010)	0.640 (0.510–0.753)
6	Moderate ARC	709	0.864 (0.006)	0.901 (0.799–0.986)	249	0.675 (0.010)	0.680 (0.550–0.800)
7	Moderate ARC + well-to-partly controlled asthma	688	0.847 (0.006)	0.900 (0.775–0.981)	252	0.668 (0.010)	0.660 (0.560–0.780)
8	Moderate ARC + uncontrolled asthma	711	0.828 (0.006)	0.886 (0.745–0.962)	240	0.647 (0.011)	0.635 (0.510–0.788)
9	Severe ARC	707	0.831 (0.006)	0.888 (0.733–0.973)	223	0.666 (0.011)	0.660 (0.550–0.780)
10	Severe ARC + well-to-partly controlled asthma	718	0.845 (0.006)	0.900 (0.774–0.981)	243	0.663 (0.010)	0.660 (0.520–0.790)
11	Severe ARC + uncontrolled asthma	712	0.812 (0.006)	0.851 (0.708–0.948)	223	0.635 (0.011)	0.610 (0.500–0.760)
12	Perennial AR	697	0.842 (0.006)	0.899 (0.763–0.977)	247	0.655 (0.010)	0.650 (0.520–0.770)
13	Perennial AR + well-to-partly controlled asthma	694	0.849 (0.006)	0.900 (0.790–0.979)	248	0.650 (0.010)	0.650 (0.520–0.750)
14	Perennial AR + uncontrolled asthma	697	0.818 (0.006)	0.852 (0.709–0.960)	245	0.638 (0.012)	0.630 (0.500–0.790)

*SE* standard error of the mean, *IQR* interquartile range

Results were largely consistent with expectations, whereby more severe health states were valued lowest, and milder health states were valued higher. However, paradoxical findings were observed in that for both severe ARC and perennial AR, health utilities were rated lower than for the same condition with comorbid well-to-partly controlled asthma. The small differences here indicate little differentiation between the comorbid and isolated cases of these conditions on an aggregate level, likely a consequence of only a sub-sample having rated both conditions.

Utilities rated by children were substantially lower than those rated by adults for all health states. As in the adult population, results were largely consistent with expectations with more severe health states valued lowest, and milder health states valued higher. Again, there were some slight inconsistencies; utility for uncontrolled asthma alone was rated as slightly poorer than for uncontrolled asthma comorbid with perennial AR, mild ARC or moderate ARC. Such issues are considered in the calculation of disutilities, which were calculated at the respondent level between respondents who rated both of the health states.

One-way ANOVAs demonstrated that most utility estimates did not change significantly whether rated by patients or non-patients with the exception that adult utilities for perennial AR were rated significantly lower by adults self-reporting allergic rhinitis experience (0.817) than those not (0.849; *p* = .042).

### Disutilities

Disutilities were calculated at the respondent level, and then averaged across the samples, excluding any respondents who valued less severe health states as lower than more severe health states. Disutilities were generally highest for changes between the mildest and most severe health states, and for relative differences between isolated health states and those with uncontrolled asthma (Table 4).

**Table 4:** Average adult and child disutilities

	Adult			Child		
	*N*	Mean (SE)	Median (IQR)	*N*	Mean (SE)	Median (IQR)
Impact of increased severity						
Mild ARC						
Moderate ARC	277	0.054 (0.005)	0.019 (0.002–0.081)	93	0.131 (0.013)	0.100 (0.030–0.205)
Severe ARC	283	0.085 (0.006)	0.047 (0.007–0.117)	69	0.121 (0.015)	0.090 (0.020–0.175)
Moderate ARC						
Severe ARC	251	0.065 (0.005)	0.030 (0.003–0.098)	74	0.101 (0.013)	0.065 (0.010–0.153)
Well-to-partly controlled asthma						
Uncontrolled asthma	293	0.091 (0.006)	0.057 (0.011–0.118)	67	0.155 (0.020)	0.090 (0.030–0.240)
Impact of comorbidity						
Mild ARC						
+ Well-to-partly controlled asthma	231	0.048 (0.006)	0.011 (0.001–0.058)	93	0.089 (0.008)	0.070 (0.025–0.140)
+ Uncontrolled asthma	272	0.070 (0.005)	0.040 (0.005–0.101)	87	0.172 (0.017)	0.130 (0.030–0.290)
Moderate ARC						
+ Well-to-partly controlled asthma	216	0.058 (0.006)	0.017 (0.000–0.087)	81	0.098 (0.011)	0.070 (0.020–0.130)
+ Uncontrolled asthma	291	0.067 (0.005)	0.033 (0.004–0.101)	86	0.116 (0.012)	0.090 (0.020–0.180)
Severe ARC						
+ Well-to-partly controlled asthma	229	0.053 (0.006)	0.013 (0.003–0.071)	69	0.094 (0.013)	0.070 (0.010–0.125)
+ Uncontrolled asthma	245	0.065 (0.005)	0.037 (0.004–0.100)	74	0.104 (0.011)	0.090 (0.020–0.153)

*SEM* standard error of the mean, *IQR* interquartile range

One-way ANOVAs revealed that, for the most part, disutilities did not differ significantly between patients and non-patients (and as such, are not reported separately), with only child disutilities comparing moderate and severe ARC that were significantly larger in children whose parents reported that they have suffered with seasonal ARC (0.135) compared with those children with no parent-reported seasonal ARC (0.073; *p* = .020).

## Discussion

The utilities and disutilities generated in this study demonstrate how living with ARC and associated health conditions impacts HRQoL. In particular, the disutilities calculated indicate how increases in severity and morbidity reduce HRQoL, with the largest disutilities observed between the mild and severe forms of ARC and between isolated ARC and ARC with comorbid uncontrolled asthma.

Mean utilities generated for symptoms of ARC during pollen season were between 0.831 (severe) and 0.880 (mild; medians 0.888–0.967) in adult populations, and lower still in paediatric populations, at between 0.666 (severe) and 0.705 (mild; medians 0.600–0.700). These adult values sit between those reported in other published literature. EQ-5D-3L trial data published in Poole et al. [15] reported peak season utility values of 0.888–0.947 in patients with moderate-to-severe grass pollen allergies, closer to the median values elicited in the current study than the mean values. Conversely, Petersen et al. [16] reported EQ-5D-3L utilities for symptomatic ARC that were much lower than those observed in the current study, at 0.70 for individuals with ARC and 0.72 for those with ARC and comorbid perennial AR. It should be noted that the trial population in Petersen et al. [16] included approximately 50% patients with comorbid asthma, which may have influenced the lower utility reported, however, the paper did not report utilities separated by presence of comorbidity. Given the absence of previously published paediatric estimates, it remains unclear whether the difference in magnitude of values elicited by adults and children reflects true differences in priorities. The possibility that they result from either methodological differences or the difficulties of conducting such studies in child populations is discussed in the Limitations section.

To our knowledge, no published studies have previously reported utilities for ARC with comorbid asthma. The magnitude of adult utility values in the present study for seasonal ARC conditions with comorbid asthma was lower than in those with no comorbidities, at between 0.812 and 0.872 (medians 0.851–0.947). Disutilities were calculated at the respondent level, between individuals presented with both the single and comorbid health states in question. Adults estimated a utility decrement of 0.048–0.070 (depending on the severity of each morbidity) associated with the presence of asthma alongside ARC. Again, the utility estimates generated by paediatric populations were considerably lower, while disutilities were higher, a pattern that was observed across the board.

Finally, utility estimates elicited for asthma in isolation were broadly in line with the limited data available, such as Briggs et al. [17], which reports mapped utility values of 0.857 for well-controlled asthma and 0.798 for ‘not well-controlled asthma’. The current study found mean adult-generated asthma utility values to be at 0.829 (median 0.884) for uncontrolled asthma, and 0.874 (median 0.949) for well-to-partly controlled asthma. Mean child-generated utilities were lower, at 0.635 (median 0.620) for uncontrolled asthma, and 0.693 (median 0.700) for well-to-partly controlled asthma.

### Clinical meaningfulness

There are no established minimally important differences for SG-elicited or VAS-elicited utility in this health area; however, using the average minimally important differences estimated for the EQ-5D and SF-6D -elicited utilities as a guide (SF-6D: 0.04; EQ-5D: 0.07; Walters and Brazier [18]), the disutility associated with increases in severity of ARC or comorbidity would be considered to represent clinically meaningful drops in HRQoL. The lowest disutility observed for the adult sample was 0.048 (the impact of comorbid well-to-partly controlled asthma on mild ARC), up to 0.091, while those for children varied between 0.089 (median 0.065) and 0.172 (median 0.130).

Moreover, comparison with a US study that generated EQ-5D-3L utility values for a wide variety of chronic conditions [19] indicates that values of 0.831–0.880 elicited for ARC in the current study by adults are in line with their values of 0.853 for allergic rhinitis and 0.853 for chronic sinusitis, similar conditions to ARC. Relative to less similar chronic conditions with utilities in this range, comparison of values suggests that ARC impacts HRQoL to a similar level as psoriasis (0.834), benign breast disease (0.852) and urinary calculi (calcium stones in the urinary tract; 0.838). The adult values elicited for ARC conditions with comorbid asthma in the current study were as low as 0.812, corresponding closely with the EQ-5D-3L utilities elicited in Sullivan and Ghushchyan [19] for respiratory system diseases (0.816) and suggesting a similar impact on HRQoL to chronic conditions such as malignant skin growths (0.812), menopausal disorders (0.817) and urinary tract disorders (0.826).

### Health economic modelling

CUA uses the concept of health utility, rather than HRQoL per se, or another measure of clinical effectiveness, in order to weight the value of the impact on HRQoL to reflect the value attributed to that impact by a particular population, commonly the general public. The current study generated sets of values from both adult and child populations, and thus provides a source of utilities that aim to reflect the priorities and preferences of the paediatric population in addition to adults. Given that parents may not accurately perceive even their own child’s HRQoL [13], the paediatric values generated here may be a more accurate reflection of the ways in which the symptoms associated with ARC and asthma impact a child’s life, and the value that such impacts hold for children. However, as always within economic modelling, sensitivity and scenario analyses should be conducted to assess the uncertainty within the model and the impact of using alternative utility and disutility estimates.

This study has generated values for a wide range of health states; however, modelling requirements may, in some cases, require aggregation of two or more health states, for example, the estimation of an overall utility value for asthma. In such cases, averages should be weighted by the proportion of the population to which each respective health state would apply.

The study sample included large numbers of respondents self-reporting suffering with ARC, and associated conditions, highlighting the widespread nature of the diseases. Estimates between patients and non-patients were very similar. Given the volume of individuals reporting experience with these conditions in the general population (and, correspondingly, the large proportion of the general population that would be excluded in samples excluding patient groups) and that patient status made few differences, utilities elicited from a general population sample including both patients and non-patients (as recruited in the current study) would likely be most reflective of the societal value of each health state.

### Limitations

In the SG task, there was a large range in the level of risk that some adult respondents indicated they were willing to accept, with some individuals suggesting they would willingly accept 100% risk of death for a treatment which had 0% chance of improving their health state. It is likely that, for some adult respondents, there was some confusion about the nature of the task and the ratings that they were making, and that for others, they were simply completing the task as quickly as possible and not giving the necessary consideration to the task at hand. Moreover, the complexity of some of the comorbid symptom profiles may have led to some respondents not absorbing all aspects of the conditions described. In particular, utility differences between ARC and well-to-partly controlled asthma compared with ARC alone were not very pronounced considering the differences in the conditions described, and it is possible that features of asthma were not attended to.

Similarly large ranges were observed in the child VAS data, and in general utility values elicited in children were much lower than those elicited in adults. It is likely that children, with less experience or knowledge of the vast array of health conditions that people can suffer from, struggle to understand the scaling and, therefore, all estimates for these health states were scaled down. Alternatively, differences in the scores may be an artefact of using different tasks in the adults and children, and indeed previous research has demonstrated elicitation of higher values using SG methodology compared with VAS [20, 21].

Exclusion of responses lower than 0.3 is likely to have removed a large proportion of unthoughtful or confused respondents, and randomisation of the order of health state presentation across participants aimed to reduce order effects, however, these issues may explain some of the inconsistencies observed. Application of this exclusion rule resulted in the exclusion of 659 children and 219 adults, indicating that a substantial number of children in particular, were providing responses that lacked face validity, and highlighting the difficulties of conducting such studies in child populations. Sensitivity analyses exploring the impact of using different exclusion criteria are reported in Supplementary Material B, and conclude that these criteria produced the best balance of including maximal respondents while excluding the majority of substandard data.

In both the adult and child data, there were some small differences in directions against expectations, however, such differences would not be considered statistically different, and likely result from the fact that patients were allocated a random 8 of the total 14 health states, therefore were not considering all conditions relative to one another. Calculation of disutilities was conducted using respondent-level data including only respondents who had been presented with the two health states in question, rather than at the aggregate level, in order to present more consistent disutilities from individuals who had had the opportunity to consider both states in question.

Electronic administration is beneficial for allowing fast collection of data from large samples, but, inevitably, some of the nuances that occur in an interview setting are lost. For example, individuals are likely to be less inclined to reflect on their decision, while this may be encouraged in an interview setting. Vignette studies are also limited in that when asked to imagine they have an illness, participants often imagine the impact of the illness at the time immediately following diagnosis, and respond to questions accordingly, whereas patients often grow accustomed to living with a condition and although their health utility may be lower at disease onset, it commonly improves as patients get used to the condition.

## Conclusion

This study provides utility and disutility values that can be used in health economic models of new treatments for AR/ARC and asthma. The disutility values calculated demonstrate how increases in severity and morbidity reduce HRQoL, and these findings, alongside research into other chronic conditions, converge to emphasise that the impact of ARC and associated conditions on patient HRQoL should not be dismissed as inconsequential, and that treatments that reduce symptom severity and risk of asthma onset are likely to provide clinically meaningful utility gains.

## Electronic supplementary material

Below is the link to the electronic supplementary material.

Supplementary material Final (DOCX 66 KB)

Final Adult Survey (DOCX 34 KB)

Final Child Survey (DOCX 306 KB)
