# Supplementary material for: Utility elicitation in adults and children for allergic rhinoconjunctivitis and associated health states
Source: Qual Life Res. 2018 Jun 8;27(9):2383–91. doi: 10.1007/s11136-018-1910-8 (PMC6132982; doi:10.1007/s11136-018-1910-8)
Supplement: Supplementary file 1 — Supplementary material Final (DOCX 66 KB) [file 11136_2018_1910_MOESM1_ESM.docx]

**Supplementary Material**

**Respondent characteristics by country**

*Adult Sample*

A total of 1,454 respondents completed the survey (UK: 362, France: 368, Germany: 359, Slovakia: 365). For each country, the age (Table SA1) and gender (Table SA2) sample stratifications broadly met the target criteria to generate samples representative of the general population in each country.

**Table SA1 Adult respondent age group by country, *n* (%)**

|  | **18-24** | **25-34** | **35-44** | **45-54** | **55-64** | **65 and over** |
| --- | --- | --- | --- | --- | --- | --- |
| **UK** | 41 (11.3%) | 64 (17.7%) | 59 (16.3%) | 66 (18.2%) | 51 (14.1%) | 81 (22.4%) |
| **France** | 41 (11.1%) | 59 (16.0%) | 63 (17.1%) | 63 (17.1%) | 59 (16.0%) | 83 (22.6%) |
| **Germany** | 33 (9.2%) | 55 (15.3%) | 55 (15.3%) | 73 (20.3%) | 59 (16.4%) | 84 (23.4%) |
| **Slovakia** | 41 (11.2%) | 70 (19.2%) | 73 (20.0%) | 58 (15.9%) | 63 (17.3%) | 60 (16.4%) |
| **ALL** | 156 (10.7%) | 248 (17.1%) | 250 (17.2%) | 260 (17.9%) | 232 (16.0%) | 308 (21.2%) |

**Table SA2 Adult respondent gender by country, *n* (%)**

|  | **Male** | **Female** |
| --- | --- | --- |
| **UK** | 174 (48.1%) | 188 (51.9%) |
| **France** | 177 (48.1%) | 191 (51.9%) |
| **Germany** | 180 (50.1%) | 179 (49.9%) |
| **Slovakia** | 177 (48.5%) | 188 (51.5%) |
| **ALL** | 708 (48.7%) | 746 (51.3%) |

Table SA3 shows that there were high levels of respondents self-reporting previously experiencing ARC, with fewer respondents self-reporting other diagnoses, given the prevalence reported in the literature. Table SA4 shows that while 30-40% of the sample were not currently employed (including student and retired populations), and a similar proportion were in managerial, administrative or professional work, a smaller proportion considered their employment to be skilled, semi-skilled or unskilled manual work.

**Table SA3 Adult respondent self-report of relevant diagnoses by country, *n* (%)**

|  | **ARC** | **AR** | **Asthma** |
| --- | --- | --- | --- |
| **UK** | 149 (41.2%) | 29 (8.0%) | 89 (24.6%) |
| **France** | 139 (37.8%) | 100 (27.2%) | 73 (19.8%) |
| **Germany** | 124 (34.5%) | 66 (18.4%) | 61 (17.0%) |
| **Slovakia** | 88 (24.1%) | 132 (36.2%) | 36 (9.9%) |
| **ALL** | 500 (34.4%) | 327 (22.5%) | 259 (17.8%) |

**Table SA4 Adult respondent employment status by country, *n* (%)**

|  | **Managerial, administrative or professional** | **Skilled, semi-skilled or unskilled manual work** | **State pensioner, student or unemployed** |
| --- | --- | --- | --- |
| **UK** | 151 (41.7%) | 91 (25.1%) | 120 (33.1%) |
| **France** | 148 (40.2%) | 55 (14.9%) | 165 (44.8%) |
| **Germany** | 149 (41.5%) | 61 (17.0%) | 149 (41.5%) |
| **Slovakia** | 134 (36.7%) | 92 (25.2%) | 139 (38.1%) |
| **ALL** | 582 (40.0%) | 299 (20.6%) | 573 (39.4%) |

*Child Sample*

A total of 1,082 children aged 8-11 years completed the survey (UK: 263, France: 273, Slovakia: 273, Germany: 273), with an average age of 9.5 years (Table SA5). As with the adults, the gender split (Table SA6) was in line with the general population in each country.

**Table SA5 Child respondent age by country**

|  | **8 years**  *n* (%) | **9 years**  *n* (%) | **10 years**  *n* (%) | **11 years**  *n* (%) | **Mean (SD)** |
| --- | --- | --- | --- | --- | --- |
| **UK** | 79 (30.0%) | 49 (18.6%) | 62 (23.6%) | 73 (27.8%) | 9.49 (1.19) |
| **France** | 78 (28.6%) | 53 (19.4%) | 72 (26.4%) | 70 (25.6%) | 9.49 (1.16) |
| **Germany** | 67 (24.5%) | 60 (22.0%) | 63 (23.1%) | 83 (30.4%) | 9.59 (1.16) |
| **Slovakia** | 85 (31.1%) | 61 (22.3%) | 72 (26.4%) | 55 (20.1%) | 9.36 (1.12) |
| **ALL** | 309 (28.6%) | 223 (20.6%) | 269 (24.9%) | 281 (26.0%) | 9.48 (1.16) |

**Table SA6 Child respondent gender by country, *n* (%)**

|  | **Male** | **Female** |
| --- | --- | --- |
| **UK** | 124 (47.1%) | 139 (52.9%) |
| **France** | 131 (48.0%) | 142 (52.0%) |
| **Germany** | 134 (49.1%) | 139 (50.9%) |
| **Slovakia** | 134 (49.1%) | 139 (50.9%) |
| **ALL** | 523 (48.3%) | 559 (51.7%) |

The number of relevant diagnoses were higher still in the child respondents, with 55% parents reporting that their child had experienced ARC (Table SA7).

**Table SA7 Child respondent parent-report of relevant diagnoses by country, *n* (%)**

|  | **ARC** | **AR** | **Asthma** |
| --- | --- | --- | --- |
| **UK** | 182 (69.2%) | 48 (18.3%) | 90 (34.2%) |
| **France** | 137 (50.2%) | 110 (40.3%) | 81 (29.7%) |
| **Germany** | 181 (66.3%) | 82 (30.0%) | 55 (20.1%) |
| **Slovakia** | 96 (35.2%) | 182 (66.7%) | 31 (11.4%) |
| **ALL** | 596 (55.1%) | 422 (39.0%) | 257 (23.8%) |

**Supplementary Material B**

**Sensitivity analyses**

The exclusion criteria used in the base case analysis led to the exclusion of a large proportion of the responses. Sensitivity analyses assessed the impact of three different exclusion criteria on the utility values produced. The three different exclusion criteria were:

1. No exclusions; values generated when all responses are included.
2. Implausible – inconsistent; specific theory-driven rules removed responses from any individual who generated a ‘0’ utility for any of the health states, and any individual who rated mild health states as having lower utility than severe health states.
3. Implausible – 0.5 cut-off; values generated with a stricter cut-off excluding respondents giving responses lower than 0.5.

Table SB1 demonstrates the number of exclusions based on these different criteria. In the adult datasets, exclusion criteria implemented in the base case resulted in the loss of data from an average of approximately 15% respondents per health state, while the inconsistency and 0.5 cut-off exclusion criteria resulted in the loss of almost a third of the data collected for each health state (33% and 31% respectively). All exclusion criteria resulted in far greater losses of child data, with approximately 60% responses lost per health state for the base case calculations. Fewer losses were observed when applying inconsistency based criteria (an average of 37% per health state), while application of 0.5 cut-off exclusion criteria resulted in the loss of an average of 83% responses per health state.

***Respondent characteristics and dataset selection***

*Adults*

Analyses were conducted to assess whether respondent characteristics were associated with inclusion in each dataset. In the adult datasets, one-way ANOVAs demonstrated no differences in completion time between those included and excluded from any of the datasets (*p*>0.05 in all cases). Chi-square tests demonstrated that adult base case selection was also independent of country, gender, hayfever status, and allergic rhinitis status (*p*>0.05 in all cases). Selection following filtering using the inconsistency based criteria was independent of country, age, hayfever status, and allergic rhinitis status (*p*>0.05 in all cases). Finally, adult selection following filtering using the 0.5 cut-off exclusion criteria was also independent of gender, hayfever status and allergic rhinitis status (*p*>0.05 in all cases).

Age, occupational status and asthma status were associated with selection to the adult base case dataset; gender, occupational status and asthma status were associated with selection to the adult inconsistency based exclusions dataset; and country, age, occupational status and asthma status were associated with selection to the adult 0.5 cut-off exclusion dataset.

**Table SB1 Numbers of respondents included each dataset**

| **No.** | 1. Health state | **Adult** | | | | **Child** | | | |
| --- | --- | --- | --- | --- | --- | --- | --- | --- | --- |
|  |  | **Base case** | **No exclusions** | **Inconsistency** | **0.5 cut-off** | **Base case** | **No exclusions** | **Inconsistency** | **0.5 cut-off** |
| **1** | Well-partly controlled Asthma | 709 | 828 | 531 | 577 | 232 | 622 | 390 | 101 |
| **2** | Uncontrolled Asthma | 710 | 837 | 544 | 584 | 227 | 620 | 378 | 95 |
| **3** | Mild ARC | 714 | 836 | 559 | 592 | 252 | 623 | 391 | 111 |
| **4** | Mild ARC + Well-partly controlled Asthma | 700 | 835 | 545 | 572 | 253 | 620 | 374 | 115 |
| **5** | Mild ARC + Uncontrolled Asthma | 714 | 836 | 542 | 585 | 250 | 617 | 360 | 106 |
| **6** | Moderate ARC | 709 | 838 | 588 | 590 | 249 | 617 | 418 | 101 |
| **7** | Moderate ARC + Well-partly controlled Asthma | 688 | 823 | 558 | 562 | 252 | 614 | 406 | 115 |
| **8** | Moderate ARC + Uncontrolled Asthma | 711 | 831 | 583 | 579 | 240 | 619 | 413 | 95 |
| **9** | Severe ARC | 707 | 835 | 529 | 573 | 223 | 619 | 370 | 102 |
| **10** | Severe ARC + Well-partly controlled Asthma | 718 | 829 | 550 | 590 | 243 | 616 | 373 | 102 |
| **11** | Severe ARC + Uncontrolled Asthma | 712 | 837 | 532 | 570 | 223 | 620 | 373 | 97 |
| **12** | Perennial AR | 697 | 822 | 578 | 563 | 247 | 616 | 406 | 101 |
| **13** | Perennial AR + Well-partly controlled Asthma | 694 | 823 | 550 | 570 | 248 | 619 | 415 | 97 |
| **14** | Perennial AR + Uncontrolled Asthma | 697 | 822 | 571 | 557 | 245 | 614 | 405 | 102 |

Adjusted standardised residuals were assessed to explore these patterns of results, with alpha levels adjusted using the Bonferroni correction method. With regards to base case selection, fewer individuals over the age of 65 (278 out of 308; *p*=0.003), in skilled, semi-skilled or unskilled manual work (232 out of 299; *p*<0.001), and with self-reported asthma diagnoses (206 out of 259; *p*=0.007), were selected than would be expected if these characteristics were unrelated to base case selection.

With regards to the inconsistency based exclusions dataset, fewer males (446 out of 708; *p*=0.003), individuals in skilled, semi-skilled or unskilled manual work (179 out of 299; *p*=0.005), and individuals with self-reported asthma diagnoses (156 out of 259; *p*=0.015), were selected than would be expected if these characteristics were unrelated to inconsistency based selection criteria.

Finally, with regard to the 0.5 cut-off exclusion dataset, fewer individuals aged 65 and over (234 out of 308, *p* =0.004) and in skilled, semi-skilled or unskilled manual work (176 out of 299; *p*<0.001), and more individuals who were state pensioners, unemployed or students (425 out of 573, *p*=0.001), were selected than would be expected if these characteristics were unrelated to 0.5 cut-off exclusion criteria selection. Although marginally fewer individuals from Slovakia (233 out of 365) or with self-reported asthma (166 out of 259) were also selected than would be expected, these differences did not reach the Bonferroni-corrected alpha levels for statistical significance (*p*=0.01 and *p*=0.04 respectively).

*Children*

In contrast with the adult datasets, one-way ANOVAs indicated that selection for the child datasets was affected by time spent completing the survey. Regarding the base case, children who were selected spent significantly less time completing the survey (*p*=0.019), while conversely, for the inconsistency based exclusions dataset, children who were selected spent marginally more time completing the survey (*p*=0.055). Duration of survey completion did not affect selection for the 0.5 cut-off exclusion dataset (*p*=0.261).

Chi-square tests demonstrated that selection for any child dataset was independent of age, gender, and hayfever status (*p*>0.05 in all cases). Selection for the child dataset following implementation of inconsistency based exclusion criteria was also independent of country, asthma status and allergic rhinitis status (*p*>0.05 in all cases).

Country and asthma status were associated with selection to both the child base case dataset, and the 0.5 cut-off exclusion dataset. In addition, selection to the child base case dataset was also associated with allergic rhinitis status.

Adjusted standardised residuals were assessed to explore these patterns of results, with alpha levels adjusted using the Bonferroni correction method. With regards to both base case and 0.5 cut-off exclusion dataset selection, more individuals from the UK (base case: 130 out of 263; *p*<0.001; 0.5 cut-off: 69 out of 263; *p*<0.001) and fewer from Slovakia (base case: 64 out of 273; *p*<0.001; 0.5 cut-off : 21 out of 273; *p*<0.001), and more children with parent-reported asthma (base case: 123 out of 257, *p*=0.001; 0.5 cut-off: 60 out of 257, *p*=0.001) were selected than would be expected if these characteristics were unrelated to base case selection. Although marginally fewer individuals with parent-reported asthma (148 out of 422) were selected to the base case dataset than would be expected if the two were unrelated, this difference did not reach the respective Bonferroni-corrected alpha levels (*p*=0.028) and should be interpreted with caution.

**Table SB2 Average utilities and utility differences between the base case and no exclusions datasets**

| **No.** | 1. Health state | **Adult** | | | | | | **Child** | | | | | |
| --- | --- | --- | --- | --- | --- | --- | --- | --- | --- | --- | --- | --- | --- |
|  |  | **Mean** | | | **Median** | | | **Mean** | | | **Median** | | |
|  |  | **Base case** | **No exclusions** | ***Difference*** | **Base case** | **No exclusions** | ***Difference*** | **Base case** | **No exclusions** | ***Difference*** | **Base case** | **No exclusions** | ***Difference*** |
| **1** | Well-partly controlled Asthma | 0.874 | 0.812 | *0.062* | 0.949 | 0.913 | *0.036* | 0.693 | 0.605 | *0.088* | 0.700 | 0.620 | *0.080* |
| **2** | Uncontrolled Asthma | 0.829 | 0.768 | *0.061* | 0.884 | 0.850 | *0.034* | 0.635 | 0.413 | *0.222* | 0.620 | 0.400 | *0.220* |
| **3** | Mild ARC | 0.880 | 0.817 | *0.063* | 0.967 | 0.947 | *0.02* | 0.705 | 0.636 | *0.069* | 0.700 | 0.660 | *0.040* |
| **4** | Mild ARC + Well-partly controlled Asthma | 0.872 | 0.804 | *0.068* | 0.947 | 0.904 | *0.043* | 0.677 | 0.564 | *0.113* | 0.690 | 0.590 | *0.100* |
| **5** | Mild ARC + Uncontrolled Asthma | 0.844 | 0.780 | *0.064* | 0.900 | 0.888 | *0.012* | 0.643 | 0.454 | *0.189* | 0.640 | 0.470 | *0.170* |
| **6** | Moderate ARC | 0.864 | 0.792 | *0.072* | 0.901 | 0.894 | *0.007* | 0.675 | 0.507 | *0.168* | 0.680 | 0.510 | *0.170* |
| **7** | Moderate ARC + Well-partly controlled Asthma | 0.847 | 0.780 | *0.067* | 0.900 | 0.885 | *0.015* | 0.668 | 0.512 | *0.156* | 0.660 | 0.520 | *0.140* |
| **8** | Moderate ARC + Uncontrolled Asthma | 0.828 | 0.762 | *0.066* | 0.886 | 0.848 | *0.038* | 0.647 | 0.442 | *0.205* | 0.635 | 0.430 | *0.205* |
| **9** | Severe ARC | 0.831 | 0.766 | *0.065* | 0.888 | 0.850 | *0.038* | 0.666 | 0.458 | *0.208* | 0.660 | 0.470 | *0.190* |
| **10** | Severe ARC + Well-partly controlled Asthma | 0.845 | 0.786 | *0.059* | 0.900 | 0.879 | *0.021* | 0.663 | 0.478 | *0.185* | 0.660 | 0.490 | *0.170* |
| **11** | Severe ARC + Uncontrolled Asthma | 0.812 | 0.751 | *0.061* | 0.851 | 0.810 | *0.041* | 0.635 | 0.413 | *0.222* | 0.610 | 0.400 | *0.210* |
| **12** | Perennial AR | 0.842 | 0.777 | *0.065* | 0.899 | 0.881 | *0.018* | 0.655 | 0.501 | *0.154* | 0.650 | 0.510 | *0.140* |
| **13** | Perennial AR + Well-partly controlled Asthma | 0.849 | 0.790 | *0.059* | 0.900 | 0.885 | *0.015* | 0.650 | 0.490 | *0.16* | 0.650 | 0.500 | *0.150* |
| **14** | Perennial AR + Uncontrolled Asthma | 0.818 | 0.756 | *0.062* | 0.852 | 0.821 | *0.031* | 0.638 | 0.418 | *0.22* | 0.630 | 0.400 | *0.230* |

***Utilities generated***

Table SB2 compares the magnitude of the mean and median utilities calculated using the base case with those calculated from a dataset with no exclusions. Values generated with no exclusions were lower across the board, affected by the inclusion of the implausibly low responses excluded in the base case. These differences were more pronounced in the child dataset, for which mean utilities were an average of 0.168 points lower (median 0.164). Differences in the adult dataset were less extreme, with mean utilities and average of 0.063 points lower (median 0.029).

Table SB3 compares the magnitude of the mean and median utilities calculated using the base case with those calculated from a dataset with inconsistency based exclusion criteria. Values generated for the inconsistency based exclusions dataset were far more similar to the base case than those in the no exclusion dataset, particularly with regards to those elicited by adults (average median difference of 0.008; mean difference of 0.027). Although the values calculated in the base case were generally higher than those in the inconsistency based exclusions dataset, this finding was not consistent across all datasets. Again, the difference between datasets elicited by children were of greater magnitude than those elicited by adults, at an average of 0.176 mean values and 0.190 for median values.

Table SB4 compares the magnitude of the mean and median utilities calculated using the base case with those calculated from a dataset with 0.5 cut-off exclusion criteria. Values generated using the 0.5 cut-off exclusion criteria were higher than those generated for the base case. This difference in adults was greater for mean values (average difference of 0.051) than for medians (0.027). Once again, this difference was more pronounced in the child dataset, with an average difference of 0.100 (mean; 0.108 median).

**Table SB3 Average utilities and utility differences between the base case and inconsistency based exclusions datasets**

| **No.** | 1. Health state | **Adult** | | | | | | | **Child** | | | | | |
| --- | --- | --- | --- | --- | --- | --- | --- | --- | --- | --- | --- | --- | --- | --- |
|  |  | **Mean** | | | | **Median** | | | **Mean** | | | **Median** | | |
|  |  | **Base case** | **Inconsistency** | ***Difference*** | **Base case** | | **Inconsistency** | ***Difference*** | **Base case** | **Inconsistency** | ***Difference*** | **Base case** | **Inconsistency** | ***Difference*** |
| **1** | Well-partly controlled Asthma | 0.874 | 0.875 | *-0.002* | 0.949 | | 0.962 | *-0.013* | 0.693 | .640 | *0.053* | 0.700 | 0.660 | *0.040* |
| **2** | Uncontrolled Asthma | 0.829 | 0.796 | *0.002* | 0.884 | | 0.872 | *0.012* | 0.635 | .363 | *0.272* | 0.620 | 0.300 | *0.320* |
| **3** | Mild ARC | 0.880 | 0.882 | *0.012* | 0.967 | | 0.979 | *-0.012* | 0.705 | .694 | *0.011* | 0.700 | 0.740 | *-0.040* |
| **4** | Mild ARC + Well-partly controlled Asthma | 0.872 | 0.870 | *0.029* | 0.947 | | 0.951 | *-0.004* | 0.677 | .604 | *0.073* | 0.690 | 0.610 | *0.080* |
| **5** | Mild ARC + Uncontrolled Asthma | 0.844 | 0.832 | *0.025* | 0.900 | | 0.904 | *-0.004* | 0.643 | .452 | *0.191* | 0.640 | 0.455 | *0.185* |
| **6** | Moderate ARC | 0.864 | 0.835 | *0.036* | 0.901 | | 0.900 | *0.001* | 0.675 | .511 | *0.164* | 0.680 | 0.510 | *0.170* |
| **7** | Moderate ARC + Well-partly controlled Asthma | 0.847 | 0.822 | *0.031* | 0.900 | | 0.900 | *0* | 0.668 | .511 | *0.157* | 0.660 | 0.510 | *0.150* |
| **8** | Moderate ARC + Uncontrolled Asthma | 0.828 | 0.792 | *0.032* | 0.886 | | 0.868 | *0.018* | 0.647 | .411 | *0.236* | 0.635 | 0.400 | *0.235* |
| **9** | Severe ARC | 0.831 | 0.800 | *0.036* | 0.888 | | 0.880 | *0.008* | 0.666 | .420 | *0.246* | 0.660 | 0.405 | *0.255* |
| **10** | Severe ARC + Well-partly controlled Asthma | 0.845 | 0.813 | *0.016* | 0.900 | | 0.898 | *0.002* | 0.663 | .432 | *0.231* | 0.660 | 0.420 | *0.240* |
| **11** | Severe ARC + Uncontrolled Asthma | 0.812 | 0.776 | *0.026* | 0.851 | | 0.829 | *0.022* | 0.635 | .360 | *0.275* | 0.610 | 0.320 | *0.290* |
| **12** | Perennial AR | 0.842 | 0.826 | *0.027* | 0.899 | | 0.899 | *0* | 0.655 | .502 | *0.153* | 0.650 | 0.500 | *0.150* |
| **13** | Perennial AR + Well-partly controlled Asthma | 0.849 | 0.823 | *0* | 0.900 | | 0.900 | *0* | 0.650 | .487 | *0.163* | 0.650 | 0.490 | *0.160* |
| **14** | Perennial AR + Uncontrolled Asthma | 0.818 | 0.791 | *0* | 0.852 | | 0.850 | *0.002* | 0.638 | .394 | *0.244* | 0.630 | 0.350 | *0.280* |

***Summary***

In adults, base case values were higher than those calculated with no exclusions, lower than those calculated with 0.5 cut-off exclusion criteria, and very similar to those calculated with the use of inconsistency based exclusion criteria (see Figure SB1). Exclusions based on specified cut-off values (base case and 0.5 cut-off) disproportionately targeted individuals over the age of 65, indicating that older respondents tended to value health states lower than other age groups. Similarly, implementation of the 0.5 cut-off exclusion criteria disproportionately targeted Slovakian respondents, indicating that more individuals from Slovakia valued health states below 0.5. It is possible that these findings may reflect the impact of a poorer state of background health in older respondents or differences in lifestyle more generally, that may result in perception of a greater impact of respiratory conditions on quality of life.

For all exclusion criteria, including those based on inconsistency rather than specified cut-off values, a disproportionately higher proportion of individuals in non-professional paid employment (skilled, semi-skilled or manual labour) were excluded. It is unclear whether this reflects true preference differences relating to the impact of respiratory disease on this type of occupation, or whether it is an artefact due to a greater proportion of respondents misunderstanding the standard gamble items, or lacking due care and attention. Likewise, a disproportionately greater proportion of adult respondents with asthma were excluded regardless of the criteria implemented. Had this pattern been observed only in cases with a specified cut-off, it could have been explained by a higher number of respondents giving particularly low valuations, however, this does not explain the presence of this pattern in the inconsistency based exclusions. We speculate that this could be the consequence of comprehension difficulties associated with complex relationships between increased asthma prevalence in individuals of low socio-economic status.

**Figure SB1 Mean adult utilities from different sensitivity analyses**

**Table SB4 Average utilities and utility differences between the base case and 0.5 cut-off exclusion datasets**

| **No.** | 1. Health state | **Adult** | | | | | | **Child** | | | | | | |
| --- | --- | --- | --- | --- | --- | --- | --- | --- | --- | --- | --- | --- | --- | --- |
|  |  | **Mean** | | | **Median** | | | **Mean** | | | | **Median** | | |
|  |  | **Base case** | **0.5 cut-off** | ***Difference*** | **Base case** | **0.5 cut-off** | ***Difference*** | **Base case** | **0.5 cut-off** | ***Difference*** | **Base case** | | **0.5 cut-off** | ***Difference*** |
| **1** | Well-partly controlled Asthma | 0.874 | 0.922 | *-0.048* | 0.949 | 0.973 | *-0.024* | 0.693 | 0.774 | *-0.081* | 0.700 | | 0.790 | *-0.090* |
| **2** | Uncontrolled Asthma | 0.829 | 0.873 | *-0.044* | 0.884 | 0.900 | *-0.016* | 0.635 | 0.756 | *-0.121* | 0.620 | | 0.760 | *-0.140* |
| **3** | Mild ARC | 0.880 | 0.925 | *-0.045* | 0.967 | 0.982 | *-0.015* | 0.705 | 0.775 | *-0.070* | 0.700 | | 0.780 | *-0.080* |
| **4** | Mild ARC + Well-partly controlled Asthma | 0.872 | 0.924 | *-0.052* | 0.947 | 0.970 | *-0.023* | 0.677 | 0.763 | *-0.086* | 0.690 | | 0.740 | *-0.050* |
| **5** | Mild ARC + Uncontrolled Asthma | 0.844 | 0.898 | *-0.054* | 0.900 | 0.938 | *-0.038* | 0.643 | 0.742 | *-0.099* | 0.640 | | 0.735 | *-0.095* |
| **6** | Moderate ARC | 0.864 | 0.908 | *-0.044* | 0.901 | 0.944 | *-0.043* | 0.675 | 0.769 | *-0.094* | 0.680 | | 0.760 | *-0.080* |
| **7** | Moderate ARC + Well-partly controlled Asthma | 0.847 | 0.896 | *-0.049* | 0.900 | 0.927 | *-0.027* | 0.668 | 0.762 | *-0.094* | 0.660 | | 0.750 | *-0.090* |
| **8** | Moderate ARC + Uncontrolled 0.780Asthma | 0.828 | 0.878 | *-0.050* | 0.886 | 0.900 | *-0.014* | 0.647 | 0.770 | *-0.123* | 0.635 | | 0.780 | *-0.145* |
| **9** | Severe ARC | 0.831 | 0.883 | *-0.052* | 0.888 | 0.901 | *-0.013* | 0.666 | 0.763 | *-0.097* | 0.660 | | 0.755 | *-0.095* |
| **10** | Severe ARC + Well-partly controlled Asthma | 0.845 | 0.892 | *-0.047* | 0.900 | 0.927 | *-0.027* | 0.663 | 0.775 | *-0.112* | 0.660 | | 0.775 | *-0.115* |
| **11** | Severe ARC + Uncontrolled Asthma | 0.812 | 0.864 | *-0.052* | 0.851 | 0.894 | *-0.043* | 0.635 | 0.751 | *-0.116* | 0.610 | | 0.760 | *-0.150* |
| **12** | Perennial AR | 0.842 | 0.893 | *-0.051* | 0.899 | 0.925 | *-0.026* | 0.655 | 0.754 | *-0.099* | 0.650 | | 0.750 | *-0.100* |
| **13** | Perennial AR + Well-partly controlled Asthma | 0.849 | 0.903 | *-0.054* | 0.900 | 0.937 | *-0.037* | 0.650 | 0.766 | *-0.116* | 0.650 | | 0.750 | *-0.100* |
| **14** | Perennial AR + Uncontrolled Asthma | 0.818 | 0.871 | *-0.053* | 0.852 | 0.896 | *-0.044* | 0.638 | 0.776 | *-0.138* | 0.630 | | 0.790 | *-0.160* |

As in adults, in children base case values were higher than those calculated with no exclusions and lower than those calculated with 0.5 cut-off exclusion criteria (see Figure SB2). Values using inconsistency based exclusion criteria were the most similar to the base case for the milder health states in children too, however for the more severe health states, utilities calculated using the inconsistency based exclusions dataset were, in many cases, as low as, or lower than, those calculated with no exclusions.

Interestingly, in the child datasets base case exclusion criteria disproportionately included respondents who spent less time on the survey, while the opposite pattern was observed for inconsistency based exclusion criteria. Given the implausibly low estimates observed for some health states in the inconsistency based exclusions dataset, we speculate that longer time on task may have been indicative of lower levels of comprehension. As in adults, the disproportionate exclusion of respondents from Slovakia for datasets with specified cut-offs indicated that Slovakian nationality was associated with elicitation of a higher proportion of values that fell below cut-offs. This is discussed above. Contrary to adults, a disproportionately *higher* proportion of respondents with parent-reported asthma were selected to the datasets with specific cut-offs. This may suggest that to some children without experience of asthma, the impact of respiratory symptoms on quality of life is perceived as more detrimental than the reality.

**Figure SB2 Mean child utilities from different sensitivity analyses**

*Conclusions*

All things considered, we consider the base case utilities to present the best balance between inclusion of the preferences of a maximal number of respondents, and the exclusion responses that are the consequence of misunderstandings or lack of due care and attention.
