# Supplementary material for: Utility elicitation in adults and children for allergic rhinoconjunctivitis and associated health states
Source: Qual Life Res. 2018 Jun 8;27(9):2383–91. doi: 10.1007/s11136-018-1910-8 (PMC6132982; doi:10.1007/s11136-018-1910-8)

You are invited take part in a study being run by York Health Economics Consortium (YHEC) and sponsored by a pharmaceutical company. We would like to know about how much you value the impact different health conditions have on quality of life.

This survey has 2 parts to it. The first part of the survey asks you some questions about yourself. These questions do not ask about any personal information, and you will not be asked to provide your name, address or date of birth, or any other information which could be linked to you.

For the second part, we would like you to imagine that you are a patient who is suffering from a selection of health conditions that affect your airways. For each question, the symptoms of the health condition we want you to imagine will be described to you. You must then imagine that you are trying to decide whether or not to have a new treatment. This treatment would completely remove all of your symptoms, but there would also be a risk that it would fail and lead to death. Each question will ask you to rate the maximum level of risk at which you would still consider the treatment worthwhile for the health condition described. There are 8 rating questions.

What is your age?

- 18-24
- 25-34
- 35-44
- 45-54
- 55-64
- 65 or over

What is your gender?

- Male
- Female

Which of the following categories best describes your occupational status?

- Managerial, administrative or professional
- Skilled, semi- or unskilled manual work
- State pensioner, unemployed or student

Do you have, or have you ever had, any of the following health conditions? (tick all that apply)

- Hayfever
- Asthma
- Allergic rhinitis

Part 2

Hayfever, also called allergic rhinoconjunctivitis, is a health condition caused by allergies to seasonal pollens. It mainly affects the nose and eyes, causing an itchy, blocked and runny nose, and, in many patients, itchy, watery and sore eyes. Depending on how bad the symptoms are, people may be able to feel better using over-the-counter anti-histamine tablets, or they may need to use steroid-based nasal sprays or eye drops. Sometimes they still have symptoms even though they take a combination of these medicines.

Some people with hayfever are also allergic to things that are around all year, such as dust mites. This is known as allergic rhinitis. These cause similar nasal symptoms, but do not usually bother the eyes as often. People usually use steroid-based nasal sprays for these allergies.

People with hayfever can also suffer asthma, a health condition which sometimes makes breathing difficult and wheezy. Asthma is usually treated by inhalers. There are three different types of inhaler:

- Fast-acting ‘reliever’ inhalers which people use when their symptoms feel really bad
- Preventative ‘controller’ inhalers which people use every day to try to avoid symptom flare ups
- Combination inhalers with corticosteroid plus a long- acting reliever for more severe asthma

Sometimes people with asthma have to go into hospital for treatment when they experience a symptom flare up, where their breathing problems suddenly feel much worse.

There are 8 questions in this section. For each question, you must imagine that you have the health condition that has been described, which will be different every time. You are deciding whether you would choose to have a new and risky treatment that would remove all of the symptoms.

Each question will ask you to indicate what the highest failure rate (or lowest success rate) could be for you to still choose to have the treatment. You should show this by to moving the slider so that the shaded bar shows the highest level of risk you would accept, and therefore the unshaded part of the bar shows the chance of successful treatment.


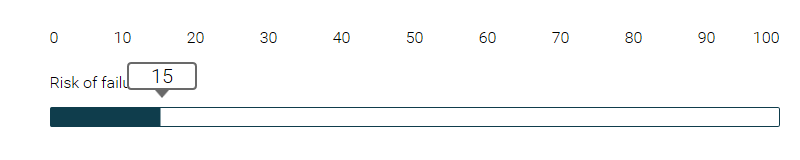


For example, if you moved the slider so that it looked how it looks in the picture above, you would be saying that you would choose to have the treatment if there was a 15% risk of failure and an 85% chance of success.

Or, if you thought you would only have the treatment for the condition described if there was a 100% success rate, you would move the slider to show 0% risk of failure, like in the picture below.


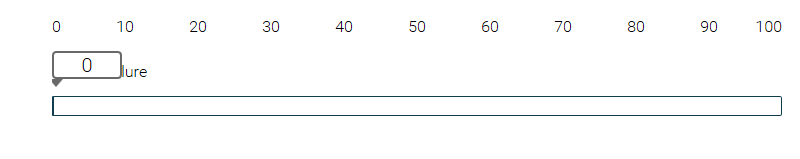


<<*please note that although all 14 health state items are presented here, respondents will be shown a maximum of 8 items each, and these will be presented in a randomized order>>*

Imagine you live with the following health condition:

*Symptoms*

- Your eyes and nose itch. Your nose is blocked and your eyes are watery. You sneeze frequently.  These symptoms are seasonal (only during pollen season).

*Impact on daily life*

- These symptoms are not very troublesome.  They do not affect your enjoyment of your free time or outdoor activities, or your ability to do daily chores or your work.

*Treatment*

- You can relieve your nasal symptoms using over-the-counter anti-histamine medications once each day.

There is a treatment available that would completely remove all of your symptoms, but may fail, which would lead to death. What is the highest risk of failure you would accept and still choose to have the treatment to remove these symptoms?


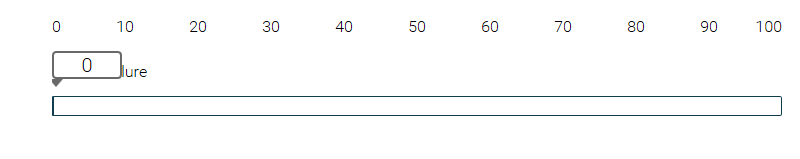


Imagine you live with the following health condition:

*Symptoms*

- Your eyes and nose are very itchy. Your nose is blocked so that you cannot breathe through it, and your eyes are watery. You sneeze frequently.  These symptoms are seasonal (only during pollen season).

*Impact on daily life*

- These symptoms are troublesome.  They affect your enjoyment of your free time, especially when outside, and your ability to do your daily chores or your work.  They also affect your sleep so that you feel tired during the day.

*Treatment*

- Your nasal symptoms do not get better with over-the-counter anti-histamine medications, so you may use a prescription steroid-based nasal inhaler twice each day. You may also use eye drops to reduce the itching in your eyes.   ​

There is a treatment available that would completely remove all of your symptoms, but may fail, which would lead to death. What is the highest risk of failure you would accept and still choose to have the treatment to remove these symptoms?


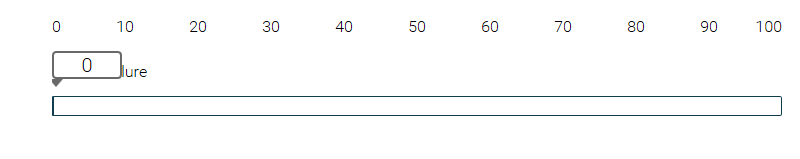


Imagine you live with the following health condition:

*Symptoms*

- Your eyes and nose are very itchy. Sometimes the itching feels unbearable. Your nose is blocked so that you cannot breathe through it, your throat is dry and your eyes are watery and sore. You sneeze frequently, several times in a row, and these outbursts mean you have to stop what you are doing.  These symptoms are seasonal (only during pollen season).

*Impact on daily life*

- These symptoms are very severe.  They affect your enjoyment of your free time, especially when outside, and your ability to do your daily chores or your work.  They also affect your sleep so that you feel tired during the day, and this can get you down.

*Treatment*

- You have to use a combination of anti-histamine medications and prescription steroid-based nasal inhalers twice each day, along with eye drops. These medications do not fully control your nasal symptoms.

There is a treatment available that would completely remove all of your symptoms, but may fail, which would lead to death. What is the highest risk of failure you would accept and still choose to have the treatment to remove these symptoms?


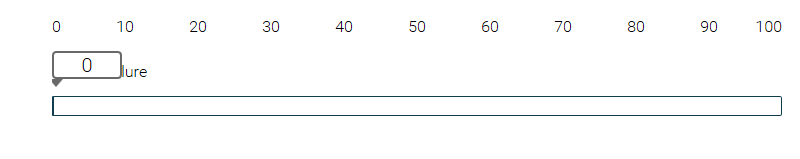


Imagine you live with the following health condition:

*Symptoms*

- Your nose is blocked so that you cannot breathe through it. You experience episodes of sneezing and/or itching and your sense of smell may be less good than it should be.  These symptoms are present all year round.

*Impact on daily life*

- These symptoms can be troublesome.  They can affect your enjoyment of your free time, both inside and outside, and may affect your ability to do your daily chores or your work. They also affect your sleep so that you feel tired during the day.

*Treatment*

- You may use a steroid-based nasal inhaler each day to try to relieve these symptoms, or take an antihistamine tablet.

There is a treatment available that would completely remove all of your symptoms, but may fail, which would lead to death. What is the highest risk of failure you would accept and still choose to have the treatment to remove these symptoms?


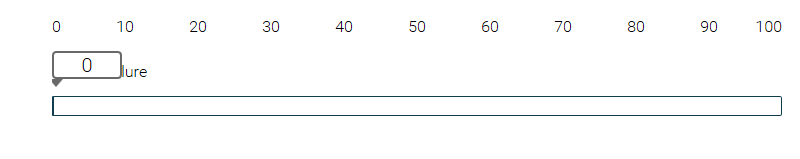


Imagine you live with the following health condition:

*Symptoms*

- Infrequently (no more than once in an average week) you experience shortness of breath, where your breathing makes a whistling sound, with chest tightness and a cough.

*Impact on daily life*

- These symptoms do not affect your ability to do physical activity, such as household chores or exercise, and do not typically affect your sleep.

*Treatment*

- You may use a preventative inhaler once or twice each day, as well as a reliever inhaler when you experience symptoms, and before exercise if this brings on the breathing problem.

There is a treatment available that would completely remove all of your symptoms, but may fail, which would lead to death. What is the highest risk of failure you would accept and still choose to have the treatment to remove these symptoms?


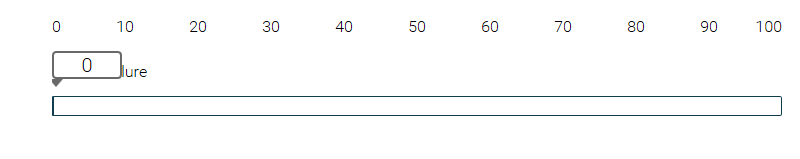


Imagine you live with the following health condition:

*Symptoms*

- More than twice a week you experience a shortness of breath, where your breathing makes a whistling sound, with chest tightness and a cough.

*Impact on daily life*

- Your breathing symptoms are worse in the evening and at night and sometimes they wake you up from your sleep.  You are limited in your ability to do physical activity, such as household chores or exercise.
- Your breathing symptoms cause you anxiety, and sometimes make you feel down.

*Treatment*

- You need to use a combination inhaler containing two different types of medicine once or twice each day, as well as a reliever inhaler when you experience symptoms, and before exercise if this brings on the breathing problem.
- Sometimes your breathing symptoms are so bad that you need to go to the hospital for treatment.

There is a treatment available that would completely remove all of your symptoms, but may fail, which would lead to death. What is the highest risk of failure you would accept and still choose to have the treatment to remove these symptoms?


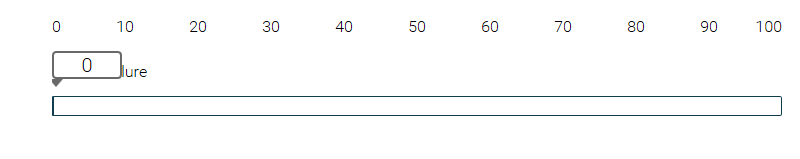


Imagine you live with the following health condition:

*Symptoms*

- Your eyes and nose itch. Your nose is blocked and your eyes are watery. You sneeze frequently.  These symptoms are seasonal (only during pollen season).
- Infrequently (no more than once in an average week) you experience shortness of breath, where your breathing makes a whistling sound, with chest tightness and a cough.

*Impact on daily life*

- Your nasal symptoms are not very troublesome. They do not affect your enjoyment of your free time or outdoor activities, or your ability to do daily chores or your work.
- Your breathing symptoms do not affect your ability to do physical activity, such as household chores or exercise, and do not typically affect your sleep.

*Treatment*

- You can relieve your nasal symptoms using over-the-counter anti-histamine medications once each day.
- For your breathing problems, you may use a preventative inhaler once or twice each day, as well as a reliever inhaler when you experience symptoms, and before exercise if this brings on the breathing problem.

There is a treatment available that would completely remove all of your symptoms, but may fail, which would lead to death. What is the highest risk of failure you would accept and still choose to have the treatment to remove these symptoms?


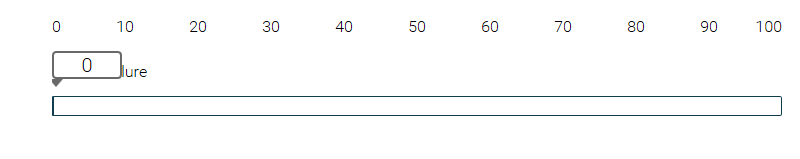


Imagine you live with the following health condition:

*Symptoms*

- Your eyes and nose itch. Your nose is blocked and your eyes are watery. You sneeze frequently.  These symptoms are seasonal (only during pollen season).
- More than twice a week you experience a shortness of breath, where your breathing makes a whistling sound, with chest tightness and a cough.

*Impact on daily life*

- Your nasal symptoms are not very troublesome. They do not affect your enjoyment of your free time or outdoor activities, or your ability to do daily chores or your work.
- Your breathing symptoms are worse in the evening and at night and sometimes they wake you up from your sleep.  You are limited in your ability to do physical activity, such as household chores or exercise.
- Your breathing symptoms cause you anxiety, and sometimes make you feel down.

*Treatment*

- You can relieve your nasal symptoms using over-the-counter anti-histamine medications once each day.
- For your breathing symptoms you need to use a combination inhaler containing two different types of medicine once or twice each day, as well as a reliever inhaler when you experience symptoms, and before exercise if this brings on the breathing problem.
- Sometimes your breathing symptoms are so bad that you need to go to the hospital for treatment.

There is a treatment available that would completely remove all of your symptoms, but may fail, which would lead to death. What is the highest risk of failure you would accept and still choose to have the treatment to remove these symptoms?


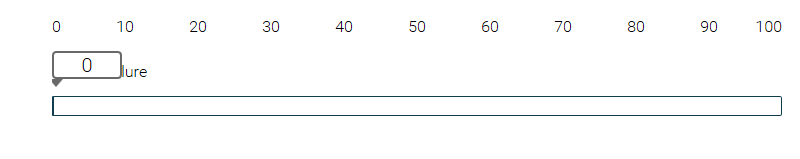


Imagine you live with the following health condition:

*Symptoms*

- Your eyes and nose are very itchy. Your nose is blocked so that you cannot breathe through it, and your eyes are watery. You sneeze frequently.  These symptoms are seasonal (only during pollen season).
- Infrequently (no more than once in an average week) you experience shortness of breath, where your breathing makes a whistling sound, with chest tightness and a cough.

*Impact on daily life*

- Your nasal symptoms are troublesome. They affect your enjoyment of your free time, especially when outside, and your ability to do your daily chores or your work.  They also affect your sleep so that you feel tired during the day.
- Your breathing symptoms do not affect your ability to do physical activity, such as household chores or exercise, and do not typically affect your sleep.

*Treatment*

- Your nasal symptoms do not get better with over-the-counter anti-histamine medications, so you may use a prescription steroid-based nasal inhaler twice each day. You may also use eye drops to reduce the itching in your eyes.
- You may use a preventative inhaler once or twice each day, as well as a reliever inhaler when you experience symptoms, and before exercise if this brings on the breathing problem.

There is a treatment available that would completely remove all of your symptoms, but may fail, which would lead to death. What is the highest risk of failure you would accept and still choose to have the treatment to remove these symptoms?


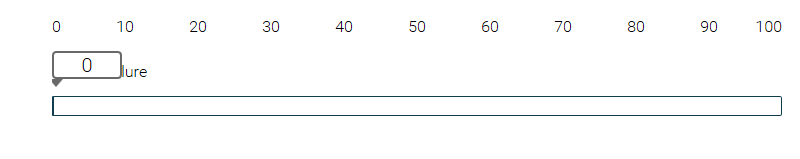


Imagine you live with the following health condition:

*Symptoms*

- Your eyes and nose are very itchy. Your nose is blocked so that you cannot breathe through it, and your eyes are watery. You sneeze frequently.  These symptoms are seasonal (only during pollen season).
- More than twice a week you experience a shortness of breath, where your breathing makes a whistling sound, with chest tightness and a cough.

*Impact on daily life*

- Your nasal symptoms are troublesome. They affect your enjoyment of your free time, especially when outside, and your ability to do your daily chores or your work.  They also affect your sleep so that you feel tired during the day.
- Your breathing symptoms are worse in the evening and at night and sometimes they wake you up from your sleep.  You are limited in your ability to do physical activity, such as household chores or exercise.
- Your breathing symptoms cause you anxiety, and sometimes make you feel down.

*Treatment*

- Your nasal symptoms do not get better with over-the-counter anti-histamine medications, so you may use a prescription steroid-based nasal inhaler twice each day. You may also use eye drops to reduce the itching in your eyes.
- You need to use a combination inhaler containing two different types of medicine once or twice each day, as well as a reliever inhaler when you experience symptoms, and before exercise if this brings on the breathing problem.
- Sometimes your breathing symptoms are so bad that you need to go to the hospital for treatment.

There is a treatment available that would completely remove all of your symptoms, but may fail, which would lead to death. What is the highest risk of failure you would accept and still choose to have the treatment to remove these symptoms?


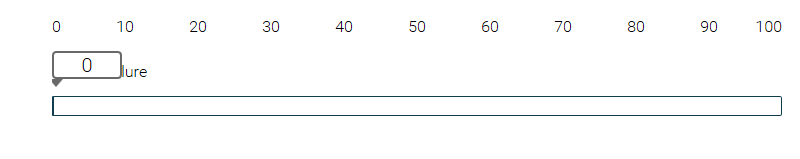


Imagine you live with the following health condition:

*Symptoms*

- Your eyes and nose are very itchy. Sometimes the itching feels unbearable. Your nose is blocked so that you cannot breathe through it, your throat is dry and your eyes are watery and sore. You sneeze frequently, several times in a row, and these outbursts mean you have to stop what you are doing.  These symptoms are seasonal (only during pollen season).
- Infrequently (no more than once in an average week) you experience shortness of breath, where your breathing makes a whistling sound, with chest tightness and a cough.

*Impact on daily life*

- Your nasal symptoms are very severe.  They affect your enjoyment of your free time, especially when outside, and your ability to do your daily chores or your work.  They also affect your sleep so that you feel tired during the day, and this can get you down.
- Your breathing symptoms do not affect your ability to do physical activity, such as household chores or exercise, and do not typically affect your sleep.

*Treatment*

- You have to use a combination of anti-histamine medications and prescription steroid-based nasal inhalers twice each day, along with eye drops. These medications do not fully control your nasal symptoms.
- For your breathing problems, you may use a preventative inhaler once or twice each day, as well as a reliever inhaler when you experience symptoms, and before exercise if this brings on the breathing problem.

There is a treatment available that would completely remove all of your symptoms, but may fail, which would lead to death. What is the highest risk of failure you would accept and still choose to have the treatment to remove these symptoms?


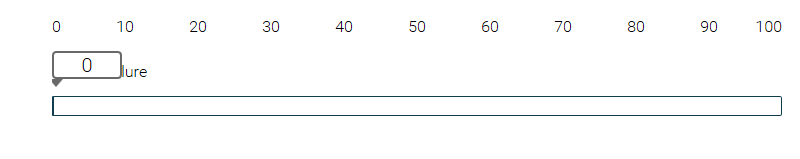


Imagine you live with the following health condition:

*Symptoms*

- Your eyes and nose are very itchy. Sometimes the itching feels unbearable. Your nose is blocked so that you cannot breathe through it, your throat is dry and your eyes are watery and sore. You sneeze frequently, several times in a row, and these outbursts mean you have to stop what you are doing.  These symptoms are seasonal (only during pollen season).
- More than twice a week you experience a shortness of breath, where your breathing makes a whistling sound, with chest tightness and a cough.

*Impact on daily life*

- Your nasal symptoms are very severe.  They affect your enjoyment of your free time, especially when outside, and your ability to do your daily chores or your work.  They also affect your sleep so that you feel tired during the day, and this can get you down.
- Your breathing symptoms are worse in the evening and at night and sometimes they wake you up from your sleep.  You are limited in your ability to do physical activity, such as household chores or exercise.
- Your breathing symptoms cause you anxiety, and sometimes make you feel down.

*Treatment*

- You have to use a combination of anti-histamine medications and prescription steroid-based nasal inhalers twice each day, along with eye drops. These medications do not fully control your nasal symptoms.
- You need to use a combination inhaler containing two different types of medicine once or twice each day, as well as a reliever inhaler when you experience symptoms, and before exercise if this brings on the breathing problem.
- Sometimes your breathing symptoms are so bad that you need to go to the hospital for treatment.

There is a treatment available that would completely remove all of your symptoms, but may fail, which would lead to death. What is the highest risk of failure you would accept and still choose to have the treatment to remove these symptoms?


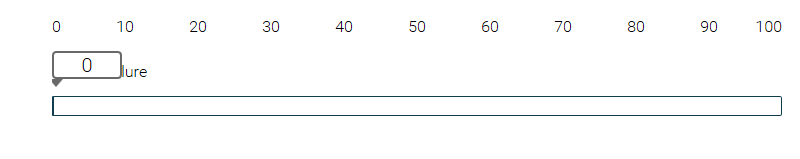


Imagine you live with the following health condition:

*Symptoms*

- Your nose is blocked so that you cannot breathe through it. You experience episodes of sneezing and/or itching and your sense of smell may be less good than it should be.  These symptoms are present all year round.
- Infrequently (no more than once in an average week) you experience shortness of breath, where your breathing makes a whistling sound, with chest tightness and a cough.

*Impact on daily life*

- Your nasal symptoms can be troublesome.  They can affect your enjoyment of your free time, both inside and outside, and may affect your ability to do your daily chores or your work. They also affect your sleep so that you feel tired during the day.
- Your breathing symptoms do not affect your ability to do physical activity, such as household chores or exercise, and do not typically affect your sleep.

*Treatment*

- For your nasal symptoms you may use a steroid-based nasal inhaler each day to try to relieve them, or take an antihistamine tablet.
- For your breathing problems, you may use a preventative inhaler once or twice each day, as well as a reliever inhaler when you experience symptoms, and before exercise if this brings on the breathing problem.

There is a treatment available that would completely remove all of your symptoms, but may fail, which would lead to death. What is the highest risk of failure you would accept and still choose to have the treatment to remove these symptoms?


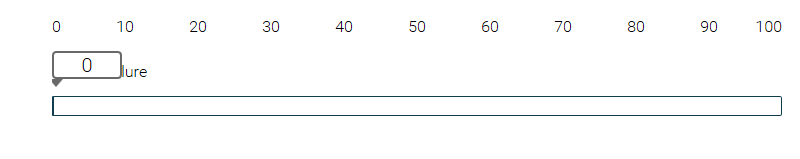


Imagine you live with the following health condition:

*Symptoms*

- Your nose is blocked so that you cannot breathe through it. You experience episodes of sneezing and/or itching and your sense of smell may be less good than it should be.  These symptoms are present all year round.
- More than twice a week you experience a shortness of breath, where your breathing makes a whistling sound, with chest tightness and a cough.

*Impact on daily life*

- Your nasal symptoms can be troublesome.  They can affect your enjoyment of your free time, both inside and outside, and may affect your ability to do your daily chores or your work. They also affect your sleep so that you feel tired during the day.
- Your breathing symptoms are worse in the evening and at night and sometimes they wake you up from your sleep.  You are limited in your ability to do physical activity, such as household chores or exercise.
- Your breathing symptoms cause you anxiety, and sometimes make you feel down.

*Treatment*

- For your nasal symptoms you may use a steroid-based nasal inhaler each day to try to relieve them, or take an antihistamine tablet.
- For your breathing symptoms you need to use a combination inhaler containing two different types of medicine once or twice each day, as well as a reliever inhaler when you experience symptoms, and before exercise if this brings on the breathing problem.
- Sometimes your breathing symptoms are so bad that you need to go to the hospital for treatment.

There is a treatment available that would completely remove all of your symptoms, but may fail, which would lead to death. What is the highest risk of failure you would accept and still choose to have the treatment to remove these symptoms?


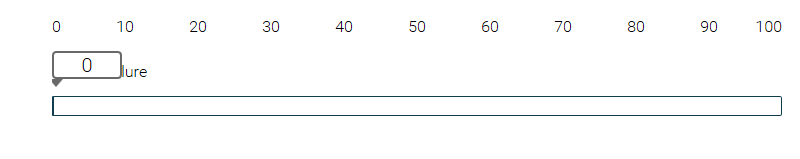

Supplement: Supplementary file 2 — Final Adult Survey (DOCX 34 KB) [file 11136_2018_1910_MOESM2_ESM.docx]
