# Supplementary material for: Utility elicitation in adults and children for allergic rhinoconjunctivitis and associated health states
Source: Qual Life Res. 2018 Jun 8;27(9):2383–91. doi: 10.1007/s11136-018-1910-8 (PMC6132982; doi:10.1007/s11136-018-1910-8)

We are inviting your child to take part in a study being run by York Health Economics Consortium, a research group from the University of York, and sponsored by a pharmaceutical company. We would like to know how much children aged between 8 and 12 years value the impact different health conditions have on quality of life.

This survey has 2 parts to it. The first part of the survey asks you some questions about your child. These questions do not ask about any personal information, and you won’t be asked to provide your child's name, address or date of birth, or any other information which could be linked to you or your child. This should only take 2 minutes of your time.

We would like your child to answer the second part. For each question, we will describe a person with a health condition that affects their airways (e.g. hayfever, allergic rhinitis, or asthma) and how it affects their life. We would then like your child to indicate how unwell they think that person is on a scale of 0 to 100. We will explain it all in a child-friendly way, but you can stay with your child and help them with the reading if you would like. We really want the ratings to show what your child thinks, so please let them decide on a number on their own. This part will take around 10 minutes for some children, but may take up to 20 minutes for children of a less advanced reading age. These answers will help us with research that will be used by organisations that make decisions about funding new types of medicine.

We will not be able to identify your child from their answers, and will look at the answers of all children altogether. Data will be accessible by the project team at YHEC and will be kept on a secure server for 5 years. All data will be handled in accordance with the Data Protection Act 1998.

Results from this study will be written in a report that we will share with the pharmaceutical company. They may also be presented at international conferences and published in academic journals. They may feature in a case study on our website.

This study was granted ethical approval by the University of York Health Sciences research Governance Committee.

If you would like more information about the study, please contact Dr Jenny Retzler on jenny.retzler@york.ac.uk. If you are unhappy about anything in this survey and wish to make a complaint, please contact Dr Matthew Taylor at matthew.taylor@york.ac.uk.

If you are happy for your child to take part in this survey, please continue with the survey. You or your child can withdraw from the survey at any time, without giving a reason, by exiting the browser window. If you exit the survey before it is finished, we may use the answers you have provided until that point.

- I have read and understood the above information and I give my consent for my child to participate in this research. I understand that they can withdraw at any time and without giving a reason.
- I do not wish to continue the survey

*<<If ‘I do not wish to continue this survey’ is selected, the survey is terminated.>>*

----------------

How old is your child?

- 8 years old
- 9 years old
- 10 years old
- 11 years old
- None of these

Is your child:

- Male
- Female

Please tick if your child has, or has ever had, any of the illnesses below? (tick all that apply)

- Hayfever
- Asthma
- Allergic rhinitis

-----------------

*<<If any of the responses above indicate that the child is ineligible, or that a quota has been filled, parents will be directed to the termination message below and will not complete the survey. >>*

I am sorry, but your child does not quite fit our requirements. It may be that we have already had lots of children of the same age or gender who have completed this survey.

Thank you for volunteering and hopefully you can help with some other research soon.

------------------

The next part is for your child. Please ask them to come and join you. You can leave them to it, or stay with them and read it through together.

-------

We are a group of scientists from York University called YHEC, and we are working with some scientists who make new types of medicine. We would like your help with some research.

Research is when a group of scientists get together to try to find something out. We are trying to find out more about what children like you think about different types of illnesses.


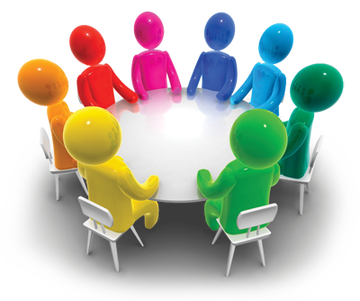


You can help us by answering some questions. There are no right or wrong answers, and if you do not want to carry on, you can stop whenever you like, and you don't need to tell us why. A grown-up can help you read the questions, but we really want to know what you think the answer should be.

---------

At some times of the year, mainly the spring and summer, pollen from plants is in the air. Pollen makes some people feel poorly because their body thinks that it is bad for them and tries to fight it. This is called hayfever.


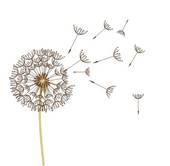


Hayfever makes people feel a bit like they have a cold. It makes their eyes and nose itchy, and it makes them sneeze. Their eyes are watery and sore, and their nose is blocked.

People with hayfever can take tablets or use a liquid medicine to make them feel better. Or, if their hayfever is really bad, sometimes people use a special type of medicine that they spray up their nose. Using eye drops can also help some people.  Sometimes they feel poorly even after using all of these medicines.

Some people with hayfever may also feel poorly because of other things that are around all year, like tiny insects that live in dust. This is called allergic rhinitis. This can make their eyes and nose itchy and their nose blocked, a bit like when they have hayfever. People mostly use a spray for their nose to feel better.

Some people with hayfever have asthma too. Asthma is an illness which sometimes makes it difficult to breathe as well as usual, and which may make them cough.

People with asthma use medicine which they take using a special object called an 'inhaler' which sprays the medicine into their mouth. There are different types of medicine that are taken using inhalers. One type needs to be taken every day and another type needs to be taken when people are feeling bad, to make it easier to breathe. Some people still find it hard to breathe with these two medicines, so they need another one as well which is stronger.

Sometimes asthma can make people feel so unwell that they need to go to hospital to get help from the doctor.

-------------

For each question, we will describe a different person with an illness like one of the ones you just read about. You need to read the description and then choose a number between 0 and 100 to show how well overall you think that person feels.

Let's think of some examples.

If somebody was so poorly they were in the hospital all the time, but still had fun doing activities with the friends and family who came to visit, we would move the slider towards the 'really unwell' side, but not quite to the bottom, so maybe at 8, like this:


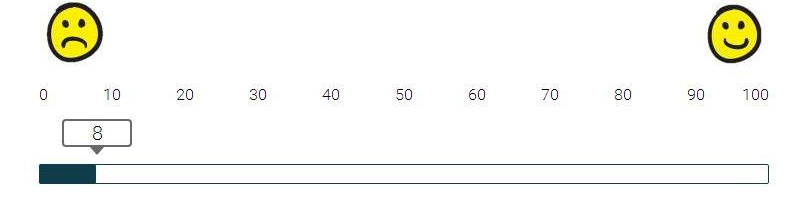


Or, if somebody was normally ok, but they had a bit of a tummy ache on one afternoon, and had to have a lie down, we might move the slider somewhere close to 100, but not quite to the top, so maybe at 95, like this:


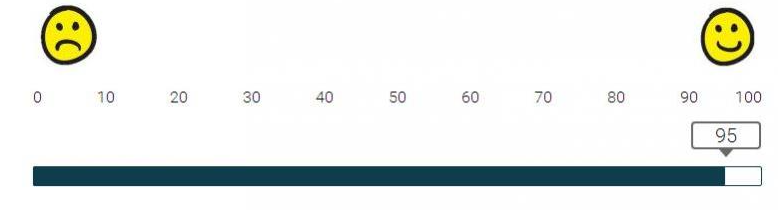


Remember, there are no right or wrong answers, and we are interested to see which number *you* think is the best.

---------------

*<<8 of the following 14 items are presented to each respondent in a randomised order. If the parents indicated that the child is male, the characters described are boys (as below). If the parents indicated the child is female, the descriptions are instead referring to girls. This is to encourage similarity to help children imagining the conditions. >>*

Alex’s eyes and nose itch. Alex’s nose is blocked and his eyes are watery. Alex sneezes a lot. Alex only feels like this at some times of the year, mainly spring and summer.

Alex can still enjoy playing and outdoor activities, and do his school work as usual.

Alex takes a tablet or liquid medicine each day to feel better.

If 0 means that someone feels the most unwell someone can ever feel, and 100 means that someone feels the most well someone can ever feel, move the slider to the number you think shows how well Alex is feeling.


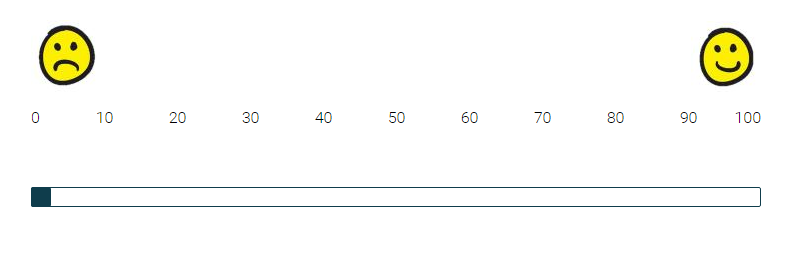


-------

Charlie’s eyes and nose itch a lot. Charlie’s nose is blocked so he can’t breathe through it, and his eyes are watery. Charlie sneezes a lot. Charlie only feels like this at some times of the year, mainly spring and summer.

It is quite annoying for him. Charlie enjoys playing less, especially outdoor activities, and feels less able do his school work. Charlie does not sleep very well, so he feels tired during the day.

Charlie uses a medicine that he sprays up his nose twice each day to feel better. Charlie also uses eye drops to make his eyes feel less sore.

If 0 means that someone feels the most unwell someone can ever feel, and 100 means that someone feels the most well someone can ever feel, move the slider to the number you think shows how well Charlie is feeling.


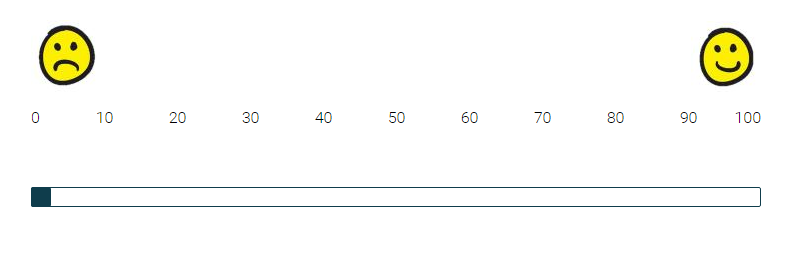


-----------

Joe’s eyes and nose itch a lot, and sometimes feel so itchy that he needs to scratch even though he shouldn’t in case it makes his eyes and nose more sore. Joe’s nose is blocked so he can’t breathe through it, and his throat is dry and eyes watery and sore. Joe sneezes lots of times in a row. These sneezes mean he has to stop what he is doing for a minute. Joe only feels like this at some times of the year, mainly spring and summer.

It is very annoying for him. Joe enjoys playing less, especially outdoor activities, and feels less able do his school work. Joe does not sleep very well, so he feels tired during the day and this can make him feel unhappy.

Joe takes a tablet or liquid medicine every day, and another medicine that he sprays up his nose twice each day to try to feel better. Joe also uses eye drops. These medicines do not make him feel completely better.

If 0 means that someone feels the most unwell someone can ever feel, and 100 means that someone feels the most well someone can ever feel, move the slider to the number you think shows how well Joe is feeling.


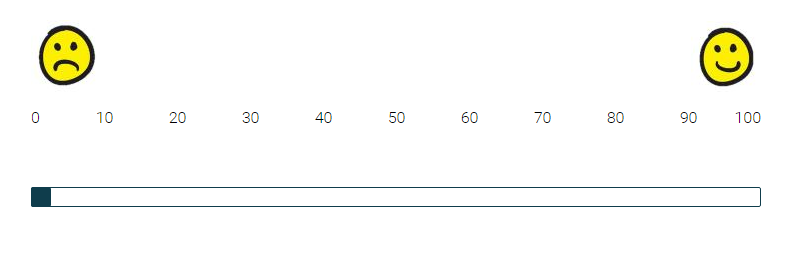


----------

Isaac’s nose is blocked so he can’t breathe through it, and he cannot smell things as well as he should. Sometimes Isaac sneezes lots of times in a row, or his eyes and nose feel itchy. Isaac feels like this all year round.

It is quite annoying for him. Isaac enjoys playing less, both indoors and outdoors, and feels less able do his school work. Isaac does not sleep very well, so he feels tired during the day.

Isaac uses a medicine that he sprays up his nose twice each day to feel better or takes a tablet or liquid medicine.

If 0 means that someone feels the most unwell someone can ever feel, and 100 means that someone feels the most well someone can ever feel, move the slider to the number you think shows how well Isaac is feeling.


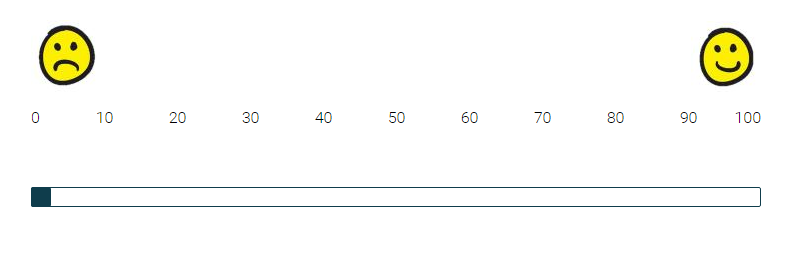


----------

No more than once a week, George feels like he can’t breathe as well as usual. George’s breathing makes a whistling sound, and his chest feels tight. This makes George cough.

This does not stop George playing or doing sports, dancing or PE, and does not make it difficult to sleep.

When George feels like this, he uses a medicine inhaler to make breathing easier. George also uses this before doing sport, dancing or PE, which can bring on the breathing problem.

If 0 means that someone feels the most unwell someone can ever feel, and 100 means that someone feels the most well someone can ever feel, move the slider to the number you think shows how well George is feeling.


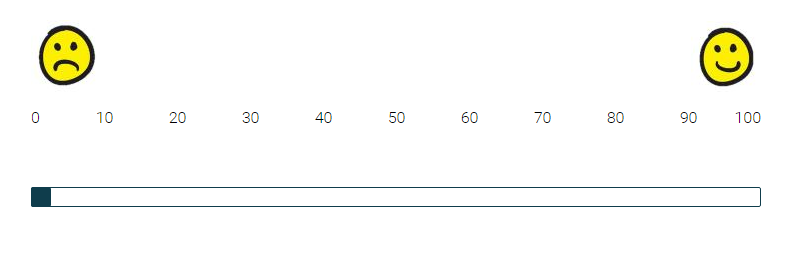


----------

More than twice a week, Oliver feels like he can’t breathe as well as usual. Oliver’s breathing makes a whistling sound, and his chest feels tight. This makes Oliver cough.

Oliver feels like this more during the evening and at night time, and sometimes he wakes up in the night feeling like this. Oliver has to be careful playing or doing sports, dancing or PE in case he has breathing problems. These breathing problems make Oliver worry and feel unhappy.

Oliver needs to use a strong medicine in an inhaler twice each day so that he doesn’t feel like this as often. When Oliver does have breathing problems, and before doing sports, dancing or PE, he uses another type of medicine inhaler to make breathing easier.

Sometimes Oliver feels so unwell he has to go to the hospital to get better.

If 0 means that someone feels the most unwell someone can ever feel, and 100 means that someone feels the most well someone can ever feel, move the slider to the number you think shows how well Oliver is feeling.


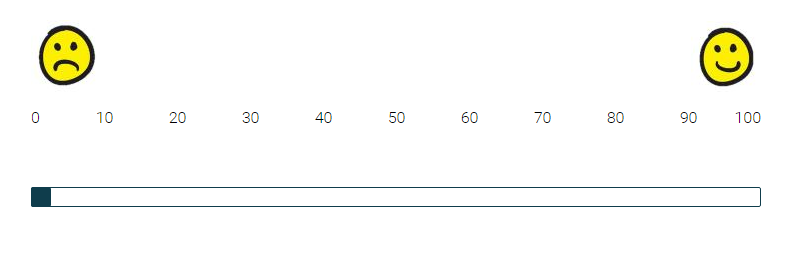


----------

Chris’ eyes and nose itch. Chris’ nose is blocked and his eyes are watery. Chris sneezes a lot. Chris only feels like this at some times of the year, mainly spring and summer.

No more than once a week, Chris feels like he can’t breathe as well as usual. Chris’ breathing makes a whistling sound, and his chest feels tight. This makes Chris cough.

Chris can still enjoy playing and outdoor activities, and do his school work as usual. The breathing problems do not stop Chris playing or doing sports, dancing or PE, and do not make it difficult to sleep.

Chris takes a tablet or liquid medicine each day to make the problems with his nose feel better.

When Chris has problems with breathing, he uses a medicine inhaler to make breathing easier. Chris also uses this before doing sport, dancing or PE, which can bring on the breathing problem.

If 0 means that someone feels the most unwell someone can ever feel, and 100 means that someone feels the most well someone can ever feel, move the slider to the number you think shows how well Chris is feeling.


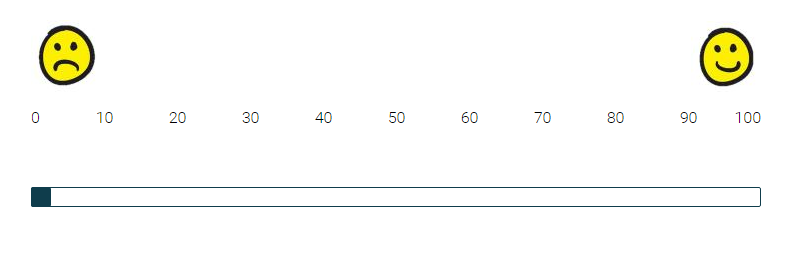


----------

Ethan’s eyes and nose itch. Ethan’s nose is blocked and his eyes are watery. Ethan sneezes a lot. Ethan only feels like this at some times of the year, mainly spring and summer.

More than twice a week, Ethan feels like he can’t breathe as well as usual. Ethan’s breathing makes a whistling sound, and his chest feels tight. This makes Ethan cough.

Ethan can still do his school work as usual. Ethan has breathing problems more often during the evening and at night time, and sometimes he wakes up in the night feeling like this. Ethan has to be careful playing or doing sports, dancing or PE in case he has breathing problems. These breathing problems make Ethan worry and feel unhappy.

Ethan takes a tablet or liquid medicine each day to make the problems with his nose feel better.

Ethan needs to use a strong medicine in an inhaler twice each day so that he doesn’t have breathing problems as often. When Ethan does have breathing problems, and before doing sports, dancing or PE, he uses another type of medicine inhaler to make breathing easier. Sometimes Ethan feels so unwell with his breathing problems he has to go to the hospital to get better.

If 0 means that someone feels the most unwell someone can ever feel, and 100 means that someone feels the most well someone can ever feel, move the slider to the number you think shows how well Ethan is feeling.


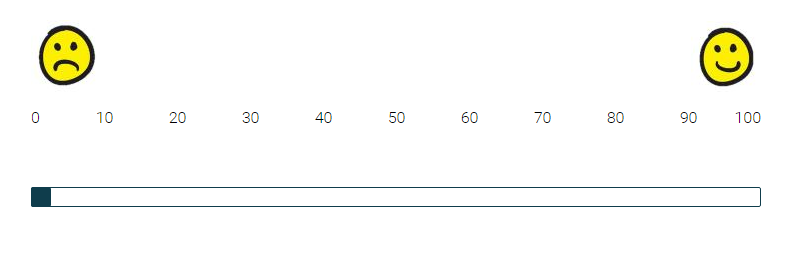


----------

Jamie’s eyes and nose itch a lot. Jamie’s nose is blocked so he can’t breathe through it, and his eyes are watery. Jamie sneezes a lot. Jamie only feels like this at some times of the year, mainly spring and summer.

No more than once a week, Jamie feels like he can’t breathe as well as usual. Jamie’s breathing makes a whistling sound, and his chest feels tight. This makes Jamie cough.

The problems with his nose and eyes are quite annoying for him. Jamie enjoys playing less, especially outdoor activities, and feels less able do his school work. Jamie does not sleep very well, so he feels tired during the day. The breathing problems do not stop Jamie playing or doing sports, dancing or PE.

Jamie uses a medicine that he sprays up his nose twice each day to make the problems with his nose feel better. Jamie also uses eye drops to make his eyes feel less sore.

When Jamie has problems with breathing, he uses a medicine inhaler to make breathing easier. Jamie also uses this before doing sport, dancing or PE, which can bring on the breathing problem.

If 0 means that someone feels the most unwell someone can ever feel, and 100 means that someone feels the most well someone can ever feel, move the slider to the number you think shows how well Jamie is feeling.


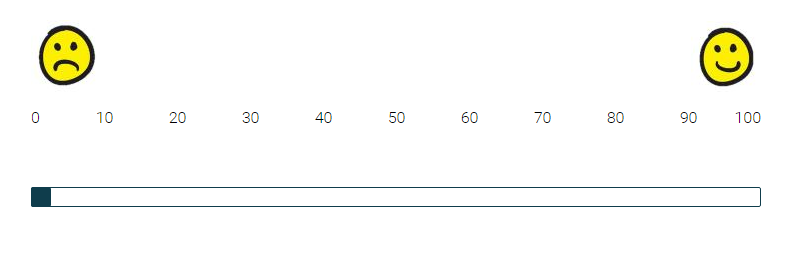


----------

Lewis’ eyes and nose itch a lot. Lewis’ nose is blocked so he can’t breathe through it, and his eyes are watery. Louis sneezes a lot. Lewis only feels like this at some times of the year, mainly spring and summer.

More than twice a week, Lewis feels like he can’t breathe as well as usual. Lewis’ breathing makes a whistling sound, and his chest feels tight. This makes Lewis cough.

The problems with his nose and eyes are quite annoying for him. Lewis enjoys playing less, especially outdoor activities, and feels less able do his school work. Lewis does not sleep very well, so he feels tired during the day.

Lewis has breathing problems more often during the evening and at night time, and sometimes he wakes up in the night feeling like this. Lewis has to be careful playing or doing sports, dancing or PE in case he has breathing problems. These breathing problems make Lewis worry and feel unhappy.

Lewis uses a medicine that he sprays up his nose twice each day to make the problems with his nose feel better. Lewis also uses eye drops to make his eyes feel less sore.

Lewis needs to use a strong medicine in an inhaler twice each day so that he doesn’t have breathing problems as often. When Lewis does have breathing problems, and before doing sports, dancing or PE, he uses another type of medicine inhaler to make breathing easier. Sometimes Lewis feels so unwell with his breathing problems he has to go to the hospital to get better.

If 0 means that someone feels the most unwell someone can ever feel, and 100 means that someone feels the most well someone can ever feel, move the slider to the number you think shows how well Lewis is feeling.


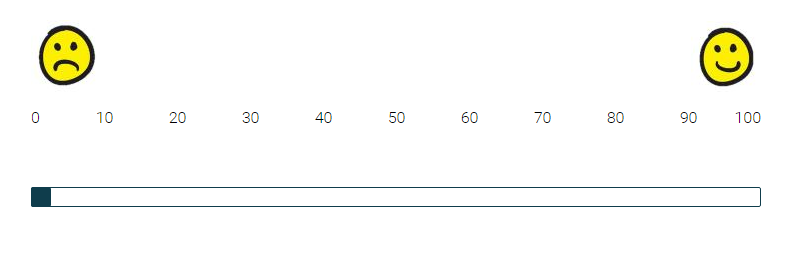


----------

Alfie’s eyes and nose itch a lot, and sometimes feel so itchy that he needs to scratch even though he shouldn’t in case it makes his eyes and nose more sore. Alfie’s nose is blocked so he can’t breathe through it, and his throat is dry and eyes watery and sore. Alfie sneezes lots of times in a row. These sneezes mean he has to stop what he is doing for a minute. Alfie only feels like this at some times of the year, mainly spring and summer.

No more than once a week, Alfie feels like he can’t breathe as well as usual. Alfie’s breathing makes a whistling sound, and his chest feels tight. This makes Alfie cough.

The problems with his nose and eye are very annoying for him. Alfie enjoys playing less, especially outdoor activities, and feels less able do his school work. Alfie does not sleep very well, so he feels tired during the day and this can make him feel unhappy. The breathing problems do not stop Alfie playing or doing sports, dancing or PE.

Alfie takes a tablet or liquid medicine every day, and another medicine that he sprays up his nose twice each day to try to make the problems with his nose better. Alfie also uses eye drops. These medicines do not make him feel completely better.

When Alfie has problems with breathing, he uses a medicine inhaler to make breathing easier. Alfie also uses this before doing sport, dancing or PE, which can bring on the breathing problem.

If 0 means that someone feels the most unwell someone can ever feel, and 100 means that someone feels the most well someone can ever feel, move the slider to the number you think shows how well Alfie is feeling.


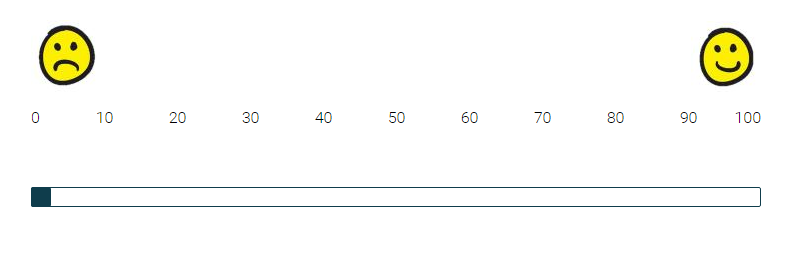
---------

Max’s eyes and nose itch a lot, and sometimes feel so itchy that he needs to scratch even though he shouldn’t in case it makes his eyes and nose more sore. Max’s nose is blocked so he can’t breathe through it, and his throat is dry and eyes watery and sore. Max sneezes lots of times in a row. These sneezes mean he has to stop what he is doing for a minute. Max only feels like this at some times of the year, mainly spring and summer.

More than twice a week, Max feels like he can’t breathe as well as usual. Max’s breathing makes a whistling sound, and his chest feels tight. This makes Max cough.

The problems with his nose and eye are very annoying for him. Max enjoys playing less, especially outdoor activities, and feels less able do his school work. Max does not sleep very well, so he feels tired during the day and this can make him feel unhappy.

Max has breathing problems more often during the evening and at night time, and sometimes he wakes up in the night feeling like this. Max has to be careful playing or doing sports, dancing or PE in case he has breathing problems. These breathing problems make Max worry and feel unhappy.

Max takes a tablet or liquid medicine every day, and another medicine that he sprays up his nose twice each day to try to make the problems with his nose better. Max also uses eye drops. These medicines do not make him feel completely better.

Max needs to use a strong medicine in an inhaler twice each day so that he doesn’t have breathing problems as often. When Max does have breathing problems, and before doing sports, dancing or PE, he uses another type of medicine inhaler to make breathing easier. Sometimes Max feels so unwell with his breathing problems he has to go to the hospital to get better.

If 0 means that someone feels the most unwell someone can ever feel, and 100 means that someone feels the most well someone can ever feel, move the slider to the number you think shows how well Max is feeling.
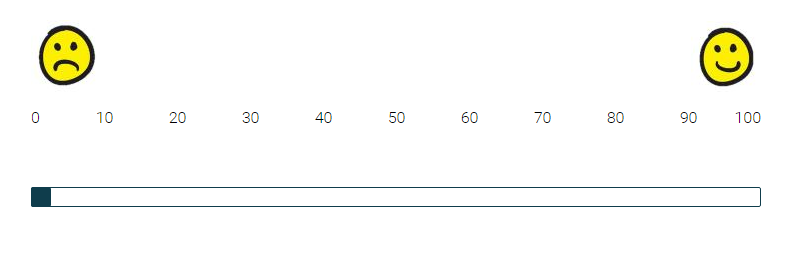


---------

Adam’s nose is blocked so he can’t breathe through it, and he cannot smell things as well as he should. Sometimes Adam sneezes lots of times in a row, or his eyes and nose feel itchy. Adam feels like this all year round.

No more than once a week, Adam feels like he can’t breathe as well as usual. Adam’s breathing makes a whistling sound, and his chest feels tight. This makes Adam cough.

The problems with his nose are quite annoying for him. Adam enjoys playing less, both indoors and outdoors, and feels less able do his school work. Adam does not sleep very well, so he feels tired during the day. The breathing problems do not stop Adam playing or doing sports, dancing or PE.

Adam uses a medicine that he sprays up his nose twice each day to make it feel better or takes a tablet or liquid medicine.

When Adam has problems with breathing, he uses a medicine inhaler to make breathing easier. Adam also uses this before doing sport, dancing or PE, which can bring on the breathing problem.

If 0 means that someone feels the most unwell someone can ever feel, and 100 means that someone feels the most well someone can ever feel, move the slider to the number you think shows how well Adam is feeling.


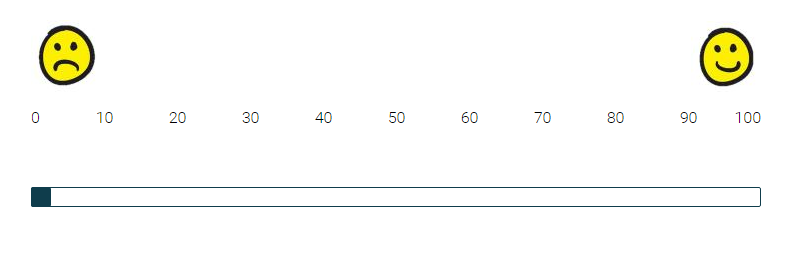


----------

Daniel’s nose is blocked so he can’t breathe through it, and he cannot smell things as well as he should. Sometimes Daniel sneezes lots of times in a row, or his eyes and nose feel itchy. Daniel feels like this all year round.

More than twice a week, Daniel feels like he can’t breathe as well as usual. Daniel’s breathing makes a whistling sound, and his chest feels tight. This makes Daniel cough.

The problems with his nose are annoying for him. Daniel enjoys playing less, both indoors and outdoors, and feels less able do his school work. Daniel does not sleep very well, so he feels tired during the day.

Daniel has breathing problems more often during the evening and at night time, and sometimes he wakes up in the night feeling like this. Daniel has to be careful playing or doing sports, dancing or PE in case he has breathing problems. These breathing problems make Daniel worry and feel unhappy.

Daniel uses a medicine that he sprays up his nose twice each day to make it feel better or takes a tablet or liquid medicine.

Daniel needs to use a strong medicine in an inhaler twice each day so that he doesn’t have breathing problems as often. When Daniel does have breathing problems, and before doing sports, dancing or PE, he uses another type of medicine inhaler to make breathing easier. Sometimes Daniel feels so unwell with his breathing problems he has to go to the hospital to get better.

If 0 means that someone feels the most unwell someone can ever feel, and 100 means that someone feels the most well someone can ever feel, move the slider to the number you think shows how well Daniel is feeling.


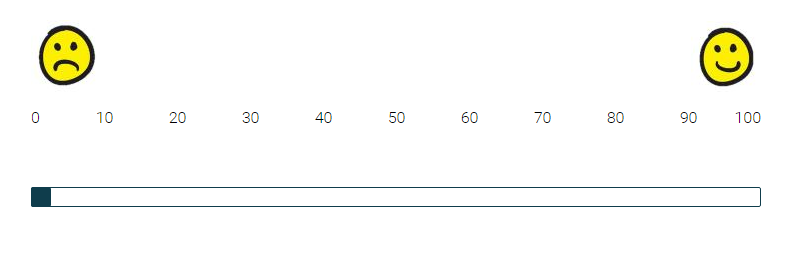

Supplement: Supplementary file 3 — Final Child Survey (DOCX 306 KB) [file 11136_2018_1910_MOESM3_ESM.docx]
